# Prognostic value of *SOX9* in cervical cancer: Bioinformatics and experimental approaches

**DOI:** 10.3389/fgene.2022.939328

**Published:** 2022-08-08

**Authors:** Huan Chen, Xupeng Chen, Fanhua Zeng, Aizhen Fu, Meiyuan Huang

**Affiliations:** ^1^ Department of Obstetrics and Gynecology, Zhu Zhou Central Hospital, Zhuzhou, Hunan China; ^2^ Laboratory Medicine Center, Zhu Zhou Central Hospital, Zhuzhou, Hunan China; ^3^ Department of Obstetrics and Gynecology, Affiliated Hospital of Guangdong Medical University, Zhanjiang, China; ^4^ Department of Pathology, Zhu Zhou Central Hospital, Zhuzhou, Hunan China

**Keywords:** differentially expressed genes (DEGs), weighted gene co-expression network analysis (WGCNA), cervical cancer, bioinformatics, hub gene, cancer-related fibroblasts

## Abstract

Among gynecological cancers, cervical cancer is a common malignancy and remains the leading cause of cancer-related death for women. However, the exact molecular pathogenesis of cervical cancer is not known. Hence, understanding the molecular mechanisms underlying cervical cancer pathogenesis will aid in the development of effective treatment modalities. In this research, we attempted to discern candidate biomarkers for cervical cancer by using multiple bioinformatics approaches. First, we performed differential expression analysis based on cervical squamous cell carcinoma and endocervical adenocarcinoma data from The Cancer Genome Atlas database, then used differentially expressed genes for weighted gene co-expression network construction to find the most relevant gene module for cervical cancer. Next, the Gene Ontology and Kyoto Encyclopedia of Genes and Genomes enrichment analyses were performed on the module genes, followed by using protein–protein interaction network analysis and Cytoscape to find the key gene. Finally, we validated the key gene by using multiple online sites and experimental methods. Through weighted gene co-expression network analysis, we found the turquoise module was the highest correlated module with cervical cancer diagnosis. The biological process of the module genes focused on cell proliferation, cell adhesion, and protein binding processes, while the Kyoto Encyclopedia of Genes and Genomes pathway of the module significantly enriched pathways related to cancer and cell circle. Among the module genes, *SOX9* was identified as the hub gene, and its expression was associated with cervical cancer prognosis. We found the expression of *SOX9* correlates with cancer-associated fibroblast immune infiltration in immune cells by Timer2.0. Furthermore, cancer-associated fibroblast infiltration is linked to cervical cancer patients’ prognosis. Compared to those in normal adjacent, immunohistochemical and real-time quantitative polymerase chain reaction (qPCR) showed that the protein and mRNA expression of *SOX9* in cervical cancer were higher. Therefore, the *SOX9* gene acts as an oncogene in cervical cancer, interactive with immune infiltration of cancer-associated fibroblasts, thereby affecting the prognosis of patients with cervical cancer.

## Introduction

Among gynecological cancers, cervical cancer (CC) is a common malignancy, which has a high mortality rate and morbidity rate (Li et al., 2020a). Its incidence is second only to breast cancer (Li et al., 2019a) and has increased significantly worldwide (Jin et al., 2020). The global incidence was estimated to be 570,000 cases and nearly 311,000 deaths in 2018 (Li et al., 2019b). In China, CC is also the second of the commonest gynecological malignancies. The incidence and mortality of it in young women are increasing (Chen et al., 2017). The pathogenesis of CC is diverse, including infection with human papillomavirus (HPV), cervical thymus (CT), herpes simplex virus type 2 (HSV-2), and other bad habits (Chen et al., 2019a). Although the pathogenesis of CC is complicated, HPV is an important reason for the development of it, which has been widely accepted (Lin et al., 2019a). The persistent infection of HPV caused by high-risk types is highly associated with CC. HPV vaccination can effectively prevent it (Gong et al., 2020). Hence, CC represents one of the most preventable cancers (Paz Soldan et al., 2008). Secondary prevention efforts such as early detection of CC and precursor lesions can reduce the incidence and mortality of it (Chen et al., 2019b). CC remains the leading cause of cancer-related death for women worldwide despite advances in prevention, detection, diagnosis, and treatment (Liu et al., 2019a), yet the molecular mechanisms underlying CC development and progression are unclear (Che et al., 2016). It is likely a multigene, multifactor, multistep, and multistage process (Zhang et al., 2014). Therefore, understanding the molecular mechanisms underlying CC pathogenesis will aid in the development of effective treatment modalities (Lin et al., 2019a).

In recent years, bioinformatics has been widely used at the genomic level of various cancer types to reveal the internal mechanisms of tumor progression and canceration (Li et al., 2020b). Molecular mechanism explanation and tumor-correlated diagnostic markers have been facilitated in part by a bioinformatics approach combining biology, mathematics, and computer science (Wang et al., 2017a). In molecular biology research, gene expression analysis plays an important role (Ye et al., 2018) and has become a popular method for detecting differentially expressed genes in human diseases (Li et al., 2020c). Weighted gene co-expression network (WGCNA) is a new tool used to identify co-expressed modules and hub genes by analyzing gene co-expression networks (Xing et al., 2020). It can be used to identify clinical biomarkers for diagnosis and therapy as it clusters highly correlated genes into one unit and relates them to clinical traits (Liu et al., 2021). As an alternative, differentially expressed genes (DEGs) from The Cancer Genome Atlas (TCGA) database were used to analyze WGCNA to identify genes associated with the occurrence and progression of cancer (Gao et al., 2020). A large-scale collaboration, TCGA, is dedicated to improving the understanding of cancer using multiple layers of genomic data (Bengtsson et al., 2009). It is a publicly available data set that provides various types of genomic data (Xu et al., 2020). At the same time, it provides detailed clinical information (Zhao et al., 2020). Therefore, it is widely used in tumor research and is useful for the identification of new biological markers and drug targets (Lin et al., 2019b).

The *SOX* protein family is a group of proteins that have a high-mobility group (HMG) domain with 50% or greater amino acid sequence similarity to that of mammalian testis-determining factor *Sry* protein. They are important in the regulation of gene expression and this activity is carried out through the conserved HMG structural domain. The family can be classified into eight different categories based on the degree of HMG sequence identity, and *SOX9* belongs to the SOXE subclass (Paskeh et al., 2021). The current study suggests that *SOX9* is a positive factor in the development of cancer; its upregulation increases cancer cell survival and metastasis; it can affect various downstream targets in cancer malignancies; upstream mediators are regulators of *SOX9* cancer; inhibition of its expression and nuclear translocation may be useful in cancer therapy (Ashrafizadeh et al., 2021).

Our study used DEGs from cervical squamous cell carcinoma and endocervical adenocarcinoma (CESC) data of TCGA to perform WGCNA and construct gene co-expression networks and tried to find key genes in the development of CC.

## Materials and methods

### Data collection

**FIGURE 1:**
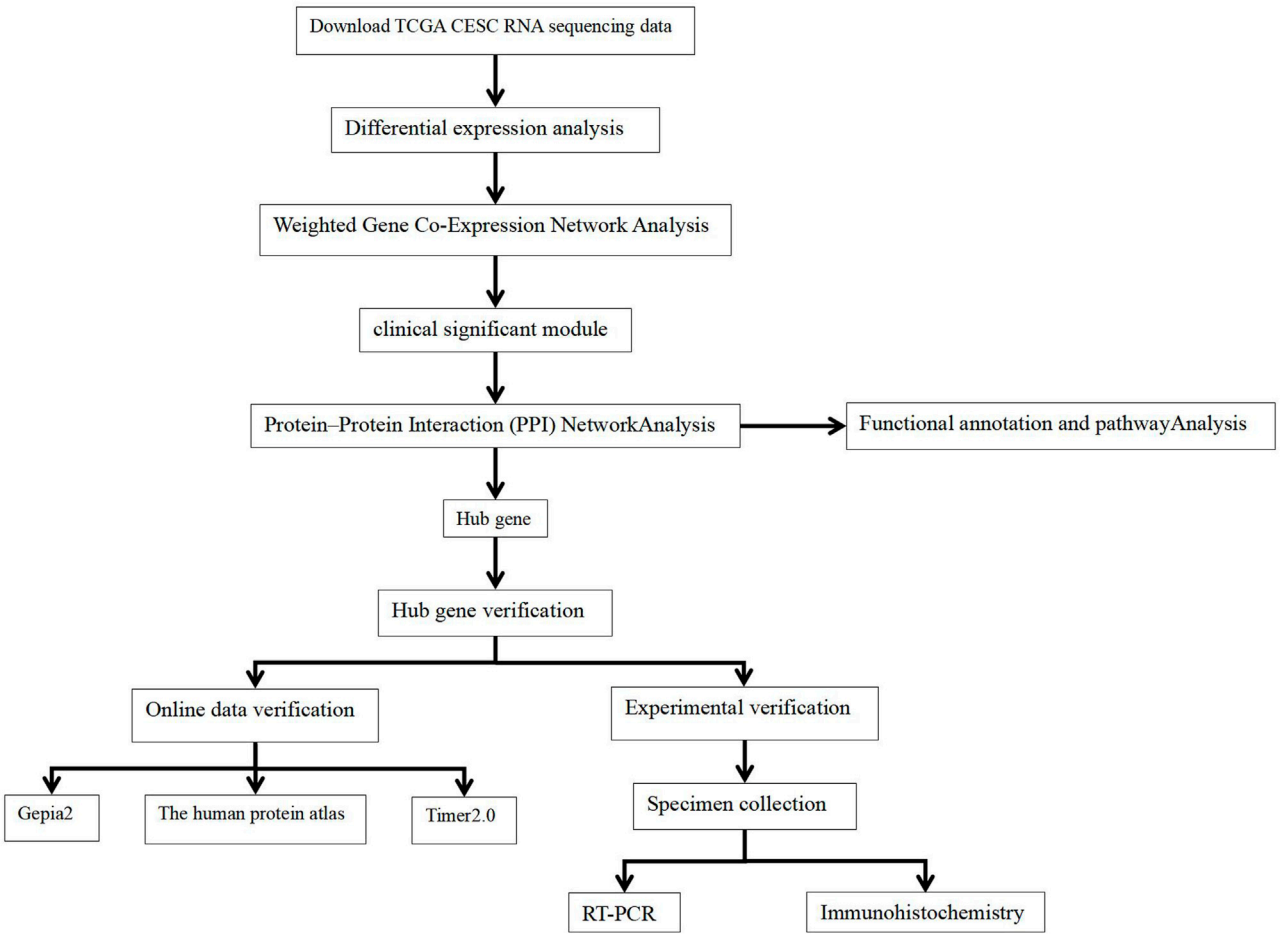
Flowchart for this study.

We obtained transcriptome data and corresponding clinical data for CECS from TCGA database (https://portal.gdc.cancer.gov/). The study included a total of 306 CESC RNA sequencing data samples and survival follow-up time and three adjacent normal tissue sequencing data samples. The workflow is illustrated in Figure 1.

### Screening for differentially expressed genes

The CESC RNA-seq counts data were downloaded from TCGA. Our studies identified DEGs by using the limma (Ritchie et al., 2015) package of R version 3.6.3 (http://www.r-project.org/). The threshold for identifying significant DEGs was FDR <0.01 and |log2 (fold change)| ≥2.

### Co-expression analysis of differentially expressed genes in carcinoma and endocervical adenocarcinoma

In systems biology, WGCNA is a widely employed method designed to analyze data with high dimensions (i.e., gene expression, DNA methylation, and metabolites) (Tremblay et al., 2019). It can reveal the correlation of genes and look for significantly related gene modules. This study constructed a co-expression network of DEGs in CECS through the “WGCNA” package (Langfelder and Horvath, 2008; Langfelder and Horvath, 2012) and analyzed potential pathogenic genes in CC.

### Identification of clinically significant modules

Modules related to clinical characteristics were identified using two methods. The gene significance (GS) was determined using the log10 transformation of the *p*-value using a linear regression analysis between gene expression and clinical characteristics, and then GS values for all genes in a module are averaged out to calculate the module significance (MS). Overall, it was generally considered that the module with the highest absolute value was most closely linked to clinical features. Furthermore, we calculated the correlation between module exigencies (MEs) and clinical traits to identify the relevant module. Further analysis was conducted on modules that were highly relevant to a given feature.

### Establishment of protein–protein interaction network and hub gene identification

We constructed a PPI network based on genes in the module which was highly relevant to a given clinical feature by using the STRING protein database 11.0 (http://string-db.org/). Next, exported and imported the results of the STRING database into the Cytoscape software (Shannon et al., 2003). The cytohubba plugin was used to find the hub genes.

### Timer2

A bioinformatics platform called TIMER2 (Li et al., 2020d) enables systematic analysis of immune infiltrates in various tumor types. It is implemented for analyzing the relationship between immune infiltration and hub gene expression (Li et al., 2020c). The expression of hub genes in various TCGA tumors and the relationship between hub genes related to immune infiltrating cells and CC prognosis were also examined.

### Pathway analysis and functional annotation

In order to identify the functional processes and pathways, DAVID’s online tool (Huang et al., 2009a; Huang et al., 2009b) was used to perform Gene Ontology (GO) and Kyoto Encyclopedia of Genes and Genomes (KEGG) analysis of genes in the module. Then, we downloaded the enrichment results and used the R software to map the results.

### Hub genes' basic expression in normal and cancer tissues

By using the gene expression profile interactive analysis (GEPIA2) database (http://gepia2.cancer-pku.cn/) (Tang et al., 2019), further verification of hub genes was performed. Both disease-free survival and the overall survival curve of hub genes were analyzed using the GEPIA online platform, with a *p*-value of <0.05. Meanwhile, we validated candidate hub genes by immunohistochemistry using the Human Protein Atlas (Uhlen et al., 2017) (https://www.proteinatlas.org/).

### Tissue collection

From October 2020 to January 2021, 16 samples of CC and adjacent normal tissues were collected at Zhuzhou Central Hospital. All specimens were evaluated by immunohistochemistry and confirmed by two independent pathologists. At the same time, fresh tissue specimens of the corresponding patients were collected and stored in an ultra-low temperature refrigerator for qPCR. Ethics committee approval was granted for the research at Zhuzhou Central Hospital (ZZCHEC2020080-01), and each eligible participant signed an informed consent form. All procedures followed the ethical guidelines laid out in the Declaration of Helsinki and relevant Chinese policies.

### Reverse transcription-quantitative polymerase chain reaction

After obtaining the hub gene, we verified the hub gene by quantitative real-time polymerase chain reaction (qPCR). RNAiso Plus (9,109; Takara, Dalian, China) was used to extract total RNA, and 1 μg of total RNA was then transcribed into complementary DNA by using the Hifair^®^ Ⅱ 1st Strand cDNA Synthesis Kit (11119ES60; Yeasen Biotechnology, Shanghai, China) according to the manufacturer’s instructions. Use this system for mRNA amplification: 85°C for 5 min, then 40 cycles of 95°C for 10 s and 60°C for 30 s. *ACTB* was used as an internal control for mRNA evaluation. To standardize *SOX9* gene expression, *ACTB* expression levels were assessed as housekeeping genes, and comparative CT (2^−ΔΔCt^) methods were used for the analysis. 2^−ΔΔCt^ indicates the ratio of target gene expression between cancer and normal groups. ΔΔCT = ΔCt cancer group - ΔCt normal group and ΔCt = Ct target gene - Ct control gene. Table1 shows the primers of *SOX9* and *ACTB.*

**TABLE 1:** Primers of *SOX9* and *ACTB*.

Primer	Sequence (5′-3′)	Product (bp)
SOX9-F2	CCC​GCT​CAC​AGT​ACG​ACT​AC	113
SOX9-R2	CTG​AGC​GGG​GTT​CAT​GTA​GG	
ACTB-F2	AGA​CCT​GTA​CGC​CAA​CAC​AG	132
ACTB-R2	CGC​TCA​GGA​GGA​GCA​ATG​AT	

### Immunohistochemistry

The following steps were followed when performing immunohistochemistry. After sections were dewaxed and hydrated, high-pressure antigen repairing was performed on them. Then, we blocked samples with goat serum and incubated them overnight with primary antibodies (Abcam ab185966 Dilution 1:800). On the next day, a secondary antibody was added, and color development was carried out by a DAB chromogen kit.

### Statistical analysis

The Student’s *t*-test (R function *t*-test) was used to determine whether there were significant differences between the two groups, and a *p*-value < 0.05 was considered to be statistically significant. The ggplot package was used for plotting.

## Result

### Differential gene expression in the cancer genome atlas carcinoma and endocervical adenocarcinoma

Based on the set criteria, there were 1,265 DEGs between 306 CESC samples and three normal samples. Select all DEGs for subsequent co-expression network construction.

### Weighted co-expression network construction

**FIGURE 2:**
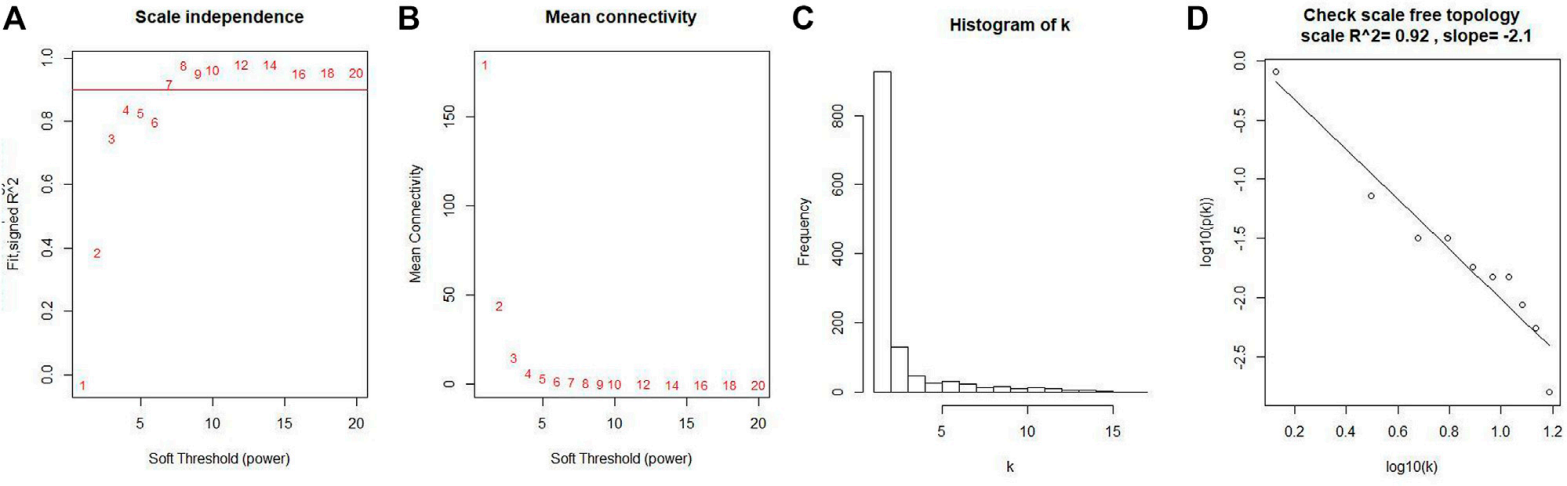
Determination of soft thresholds and testing of scale-free networks. **(A)** Correlation between log (K) and log [P (k)] corresponding to different soft thresholds. The higher the coefficient, the more the network conforms to the distribution of the scale-free network. **(B)** Mean value of gene neighbor coefficients in the gene network corresponding to different soft thresholds, which reflects the average connectivity level of the network. **(C)** Distribution of the connectivity of each node in the network. **(D)** Scatter plot of log (K) vs. log [P (k)] and the linear regression results show that the correlation coefficient is 0.92.

**FIGURE 3:**
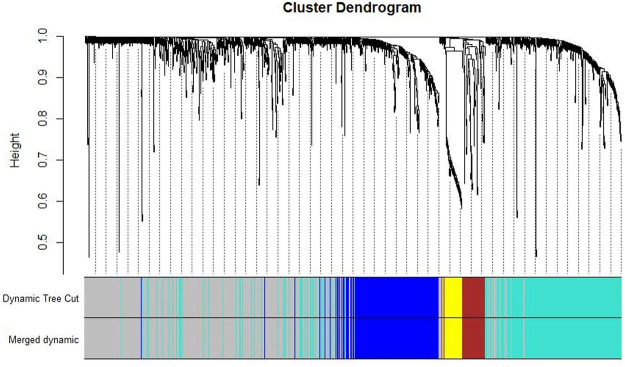
Classification of gene clustering trees by dynamic tree cut. A total of five gene modules were obtained, with different colors indicating different gene modules. The gray color indicates the genes that do not belong to any known module.

Co-expression modules were constructed with all DEGs using the WGCNA algorithm. With a soft threshold of β = 7 (ratio R2 = 0.92) (Figures 2A, B), five modules were found; Distribution of the connectivity of each node and scale free of DEGs were shown in Figures 2C,D; the minimum size (genome) of the gene tree diagram was 30, and the tangent line of the module tree diagram was 0.25 (Figure 3).

### Identification of clinically significant module

**FIGURE4:**
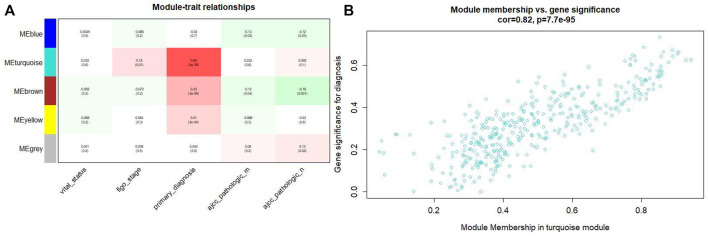
**(A)** Relationship between the module traits and the module significance. **(B)** The turquoise module has the highest correlation with the diagnosis level (*p* = 0.82, *p* = 0.65 × 3e-38).

Combining the relationship between the module traits and the module significance, we found that the turquoise module was the most relevant module for CC diagnosis. The turquoise module holds the highest correlation with the diagnosis (Figure 4). In the following analyses, the module was chosen as the module of interest.

### Gene ontology and kyoto encyclopedia of genes and genomes pathway enrichment

**FIGURE 5:**
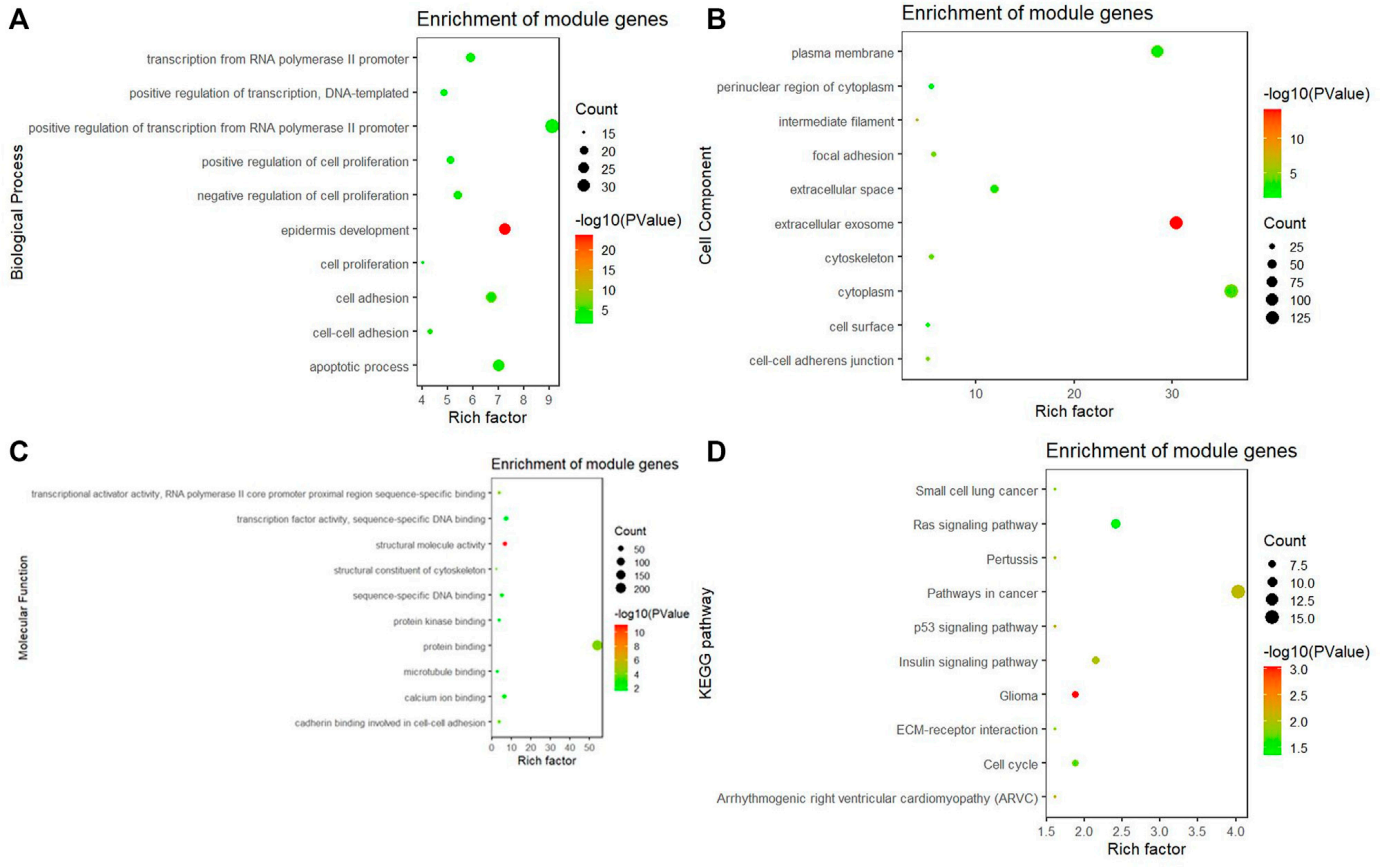
GO and KEGG pathway analysis. **(A)** Biological process of GO, **(B)** cellular components of GO, **(C)** molecular function of GO, and **(D)** KEGG pathway of the turquoise module significantly enriches pathways related to cancer and cell circle.

In the turquoise module, a total of 384 genes were enriched in GO and KEGG pathway analysis by using the ClusterProfiler and the org. Hs.eg.db packages. The biological process of the turquoise module focused on cell proliferation, cell adhesion, and protein binding processes (*p* < 0.05). The KEGG pathway of the turquoise module significantly enriched pathways related to cancer and cell circle (Figures 5A, B–D).

### PPI network construction

A PPI network was constructed in order to distinguish hub genes from common genes. Then, PPI results were analyzed by Cytoscape software. Use the cytohubba plugin in Cytoscape to estimate the node degree. Ten most critical node degree genes were selected as hub genes, and they were *CDKN2A*, *KRT5*, *SOX2*, *KRT8*, *CAV1*, *EPCAM*, *FOXA1*, *KRT14*, *ARF6*, and *SOX9*.

### Identification of hub genes and related immune infiltration cell

**FIGURE 6:**
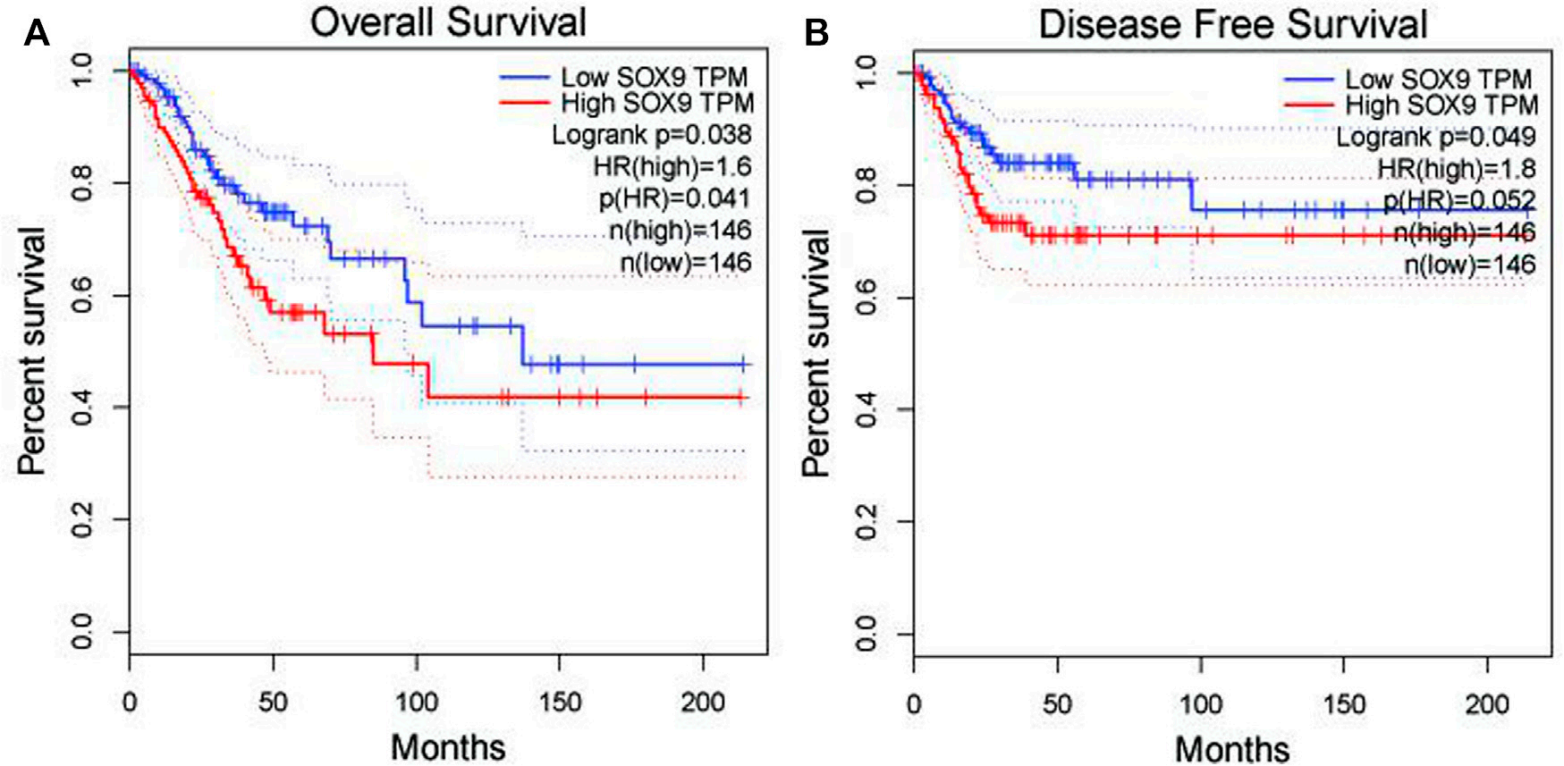
Association between *SOX9* gene expression and overall survival and disease-free survival of cervical cancer patients in TCGA. **(A)** Low *SOX9* gene expression patient had a high overall survival rate (*p* = 0.038). **(B)** Low *SOX9* gene expression patient had a high disease-free survival rate (*p* = 0.049). Low expression of *SOX9* may lead to higher survival rates and disease-free survival.

**FIGURE 7:**
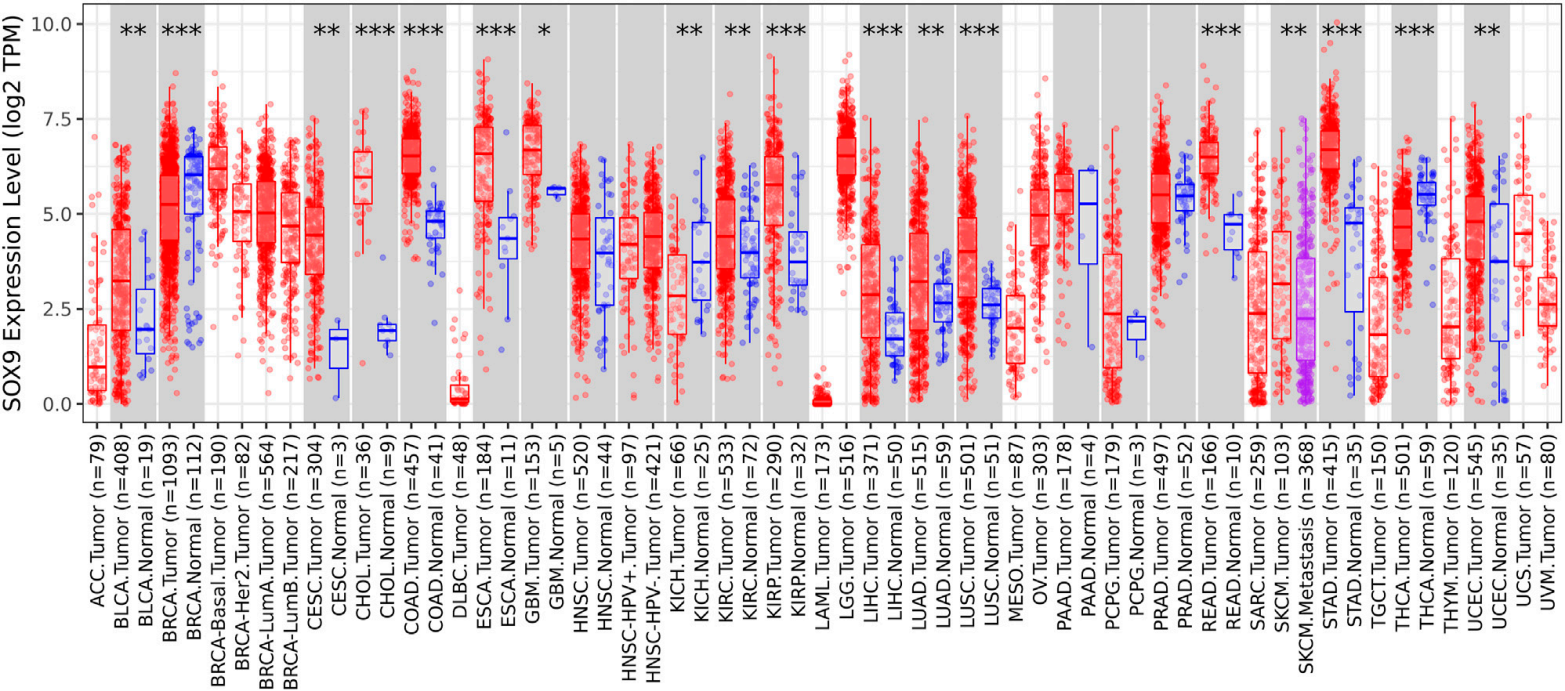
Box plot of *SOX9* expression in TCGA tumors. The mRNA expression of *SOX9* is higher than that of normal tissues in most TCGA tumors.

**FIGURE 8:**
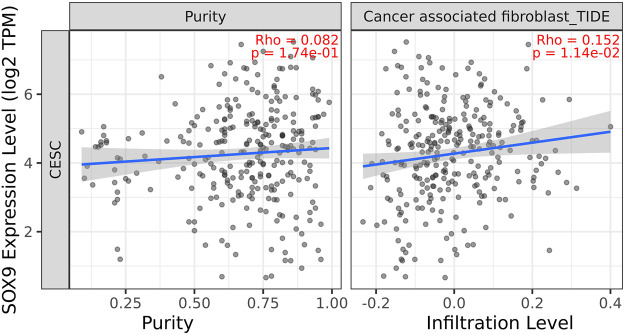
Correlation between SOX9 gene expression and CAF immune infiltration. Expression of *SOX9* positively correlates with tumor-associated fibroblasts.

**FIGURE 9:**
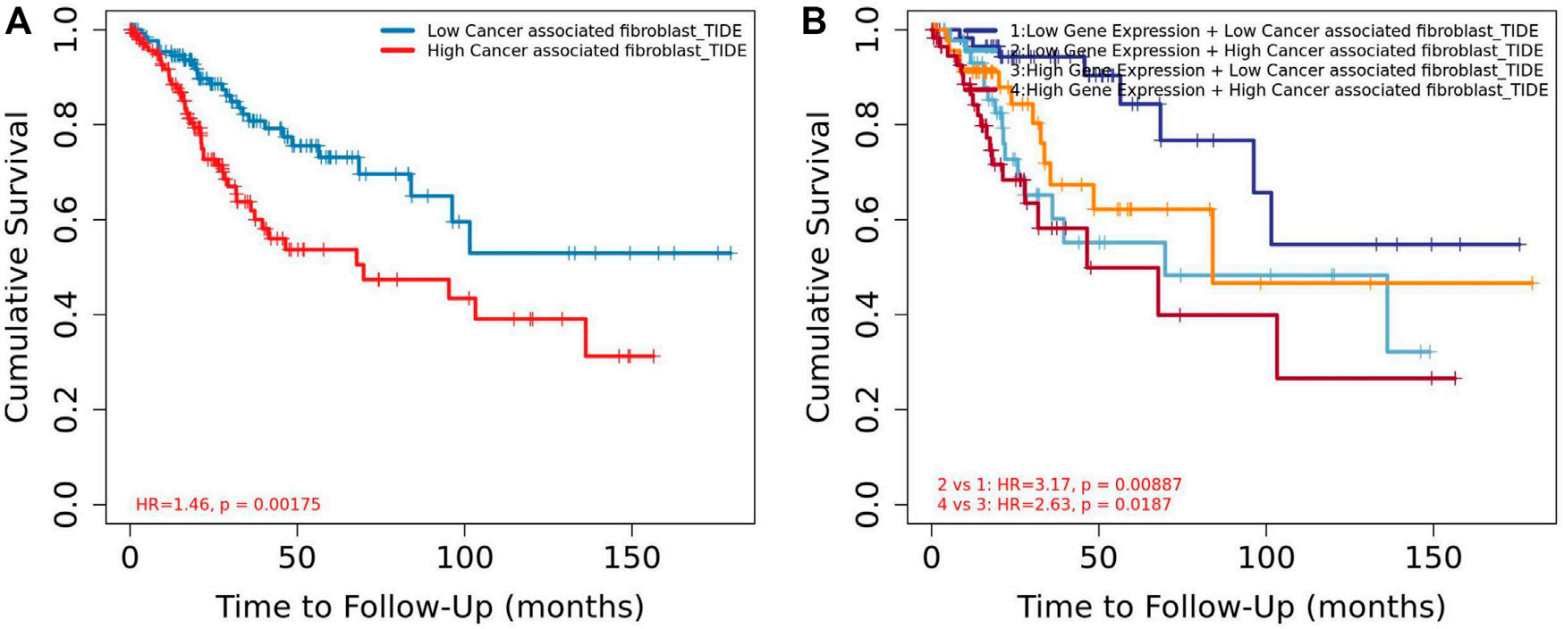
Association between CAF and overall survival in Timer2. **(A)** Low CAF had high overall survival rate than high CAF. **(B)** Low CAF + low *SOX9* expression group had high overall survival rate than the high CAF + high *SOX9* expression group.

**FIGURE 10:**
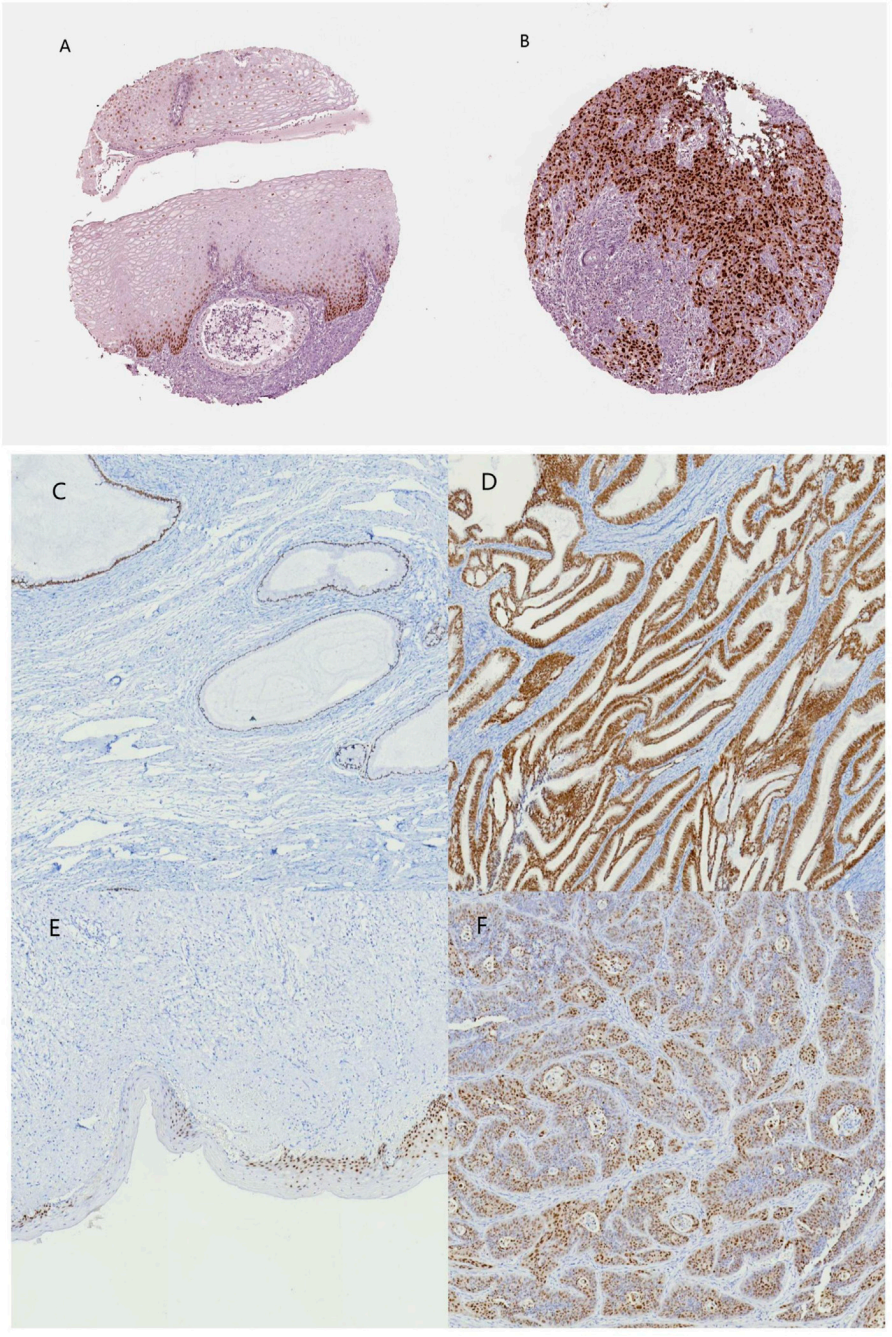
*SOX9* immunohistochemical images of normal cervical and cervical cancer tissues on the HPA database and patient samples. **(A)** normal cervix (HPA), **(B)** cervical cancer (HPA), **(C)** normal cervical glands, **(D) **cervical adenocarcinoma, **(E)** normal cervical epithelium, and **(F)** cervical squamous carcinoma.

**FIGURE 11:**
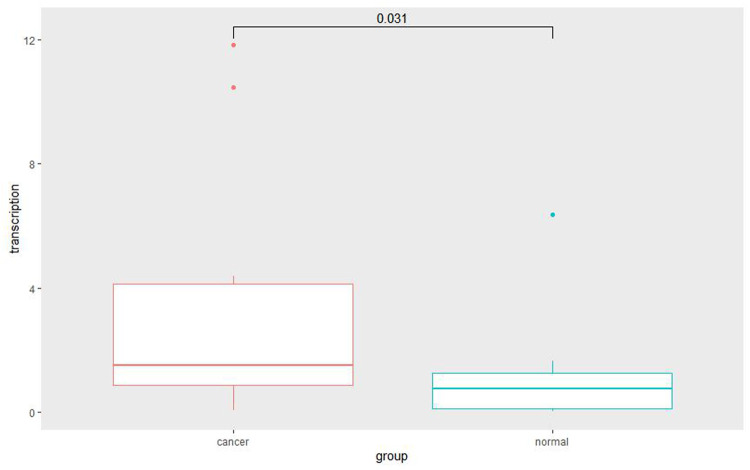
Quantitative RT-PCR for cancer and adjacent normal cervix tissue. Quantitative RT-PCR assay showed significantly increased *SOX9* mRNA level in cervical cancer tissues compared with adjacent normal cervix tissues (*p* = 0.031).

Among the 10 most important node degree genes, univariate analysis showed that *SOX9* expression level was significantly related to the overall survival (OS) and disease-free survival (DFS) of CC (*p* < 0.05) (Figures 6A, B). The mRNA expression of *SOX9* is higher than that in normal tissues in most TCGA tumors (Figure 7). The expression of *SOX9* correlates with cancer-associated fibroblast (CAF) immune infiltration in immune cells (Figure 8). Furthermore, CAF infiltration is associated with CC patients’ prognosis (Figures 9A, B). As shown in Figures 10A and B, in CC specimens, the protein expression of *SOX9* was higher than that in normal tissues in the HPA database. Similarly, the expression of *SOX9* between cancerous tissue and normal paracancerous tissue in the patient’s specimen showed the same result (Figures 10C–F). In addition, the qPCR results showed that the *SOX9* mRNA expression levels in CC tissues were upregulated compared to those in adjacent normal tissues (Figure11).

## Discussion

In this study, we downloaded the TCGA CESC mRNA count databases for differential expression analysis, then utilized the DEGs to construct a weighted co-expression network. The turquoise module was found to be closely related to the diagnosis of CC. After PPI network construction for the genes in the turquoise module, we used the Cytoscape software to calculate the results of the PPI network analysis and obtained 10 particularly important genes. Among them, *SOX9* was correlated with the overall survival and disease-free survival of CC. Compared to normal tissues, the *SOX9* mRNA expression in cancer tissues was significantly upregulated. The expression of *SOX9* correlates with CAF immune infiltration in immune cells. Furthermore, CAF infiltration is associated with CC patients’ prognosis. Also, in the Human Protein Atlas online database, the *SOX9* protein expression of CC tissues was significantly higher than that of normal tissues. Therefore, we selected the *SOX9* gene as the hub gene and collected specimens to verify our findings. Compared to adjacent normal tissues, both qPCR and immunohistochemistry results showed that *SOX9* expression was significantly higher in CC tissues.

The official name of *SOX9* is SRY-box transcription factor 9, located at 17q24.3 in humans. This transcription factor has a high-mobility group (HMG)-box DNA-binding domain that recognizes (A/T) (CA)A (T/A)G DNA sequences and controls the expression of target genes (Yamashita et al., 2019). The function of *SOX9* was first found to play a critical role in cartilage and testes development (Lei et al., 2018). Later, with the increase of research, it was found that it enhances the activity of DNA-binding transcription factors (RNA polymerase II specificity), protein binding, etc. It is also involved in many processes, including positive regulation of epithelial cell proliferation, epithelial cell migration, and cell population proliferation (Shefchek et al., 2020). *SOX9* plays an important role in many tumor types (Drivdahl et al., 2004; de Bont et al., 2008; Galmiche et al., 2008; Jiang et al., 2010; Zhou et al., 2011; Burnichon et al., 2012; Guo et al., 2012; Matheu et al., 2012; Wang et al., 2012; Zhou et al., 2012; Cai et al., 2013; Sakamoto et al., 2013; Yu et al., 2013; Wang et al., 2015a; Matsushima et al., 2015; Pomp et al., 2015; Wang et al., 2017b; Chang et al., 2017; Liu et al., 2017; Wan et al., 2017; Kim et al., 2018; Tsuda et al., 2018; Wang et al., 2018; Zhang et al., 2018; Huang et al., 2019; Yang et al., 2019; Ma et al., 2020; Voronkova et al., 2020) and acts as an oncogene in most tumors. *SOX9* could be an emerging target for anticancer drugs and a prognostic biomarker for cancer drug resistance (Tripathi et al., 2022).

The role of *SOX9* in CC has been much studied but remains controversial. Some studies suggest that *SOX9* is an oncogene in CC. *SOX9* could enhance squamous cell carcinoma cells’ colony-forming activity. Liu et al. (2016) found that *SOX9* expression in CC was higher than that in normal cervical tissue; downregulation of S*OX9* could inhibit CC cell growth and metastasis *in vivo* (Liu et al., 2019b). Studies have shown a number of molecular pathways involved in the progression of cervical cancer by targeting *SOX9*. For example, upregulation of miR-215-3p inhibited *SOX9* expression, thereby suppressing the growth and metastasis of CC cells *in vivo* (Liu et al., 2019b); *SOX9* could promote the expression of miR-130a by binding to its promoter region, thereby suppressing the expression of Ctrl and PTEN, the downstream genes of miR-130a, and ultimately increasing chemoresistance to DDP in CC cells, while a significant upregulation of *SOX9* and miR-130a expression was found in DDP-resistant CC tissues (Feng et al., 2018); Zhao et al. (2021) also demonstrated that *SOX9* could cooperate with *EGR1* to promote CC cell proliferation and invasion by inducing CC stemness, and the 5-year OS rate of low *SOX9* expression patients was significantly higher than those with high expression; He et al. (2022) found the effects of the SOX9/lncRNA ANXA2P2/miR-361-3p/SOX9 regulatory loop on cervical cancer cell growth and resistance to cisplatin. Therefore, upregulation of *SOX9* is involved in cervical cancer formation, chemoresistance, tumor cell stemness, metastasis, and invasion. All these studies suggest that *SOX9* is an oncogene in CC. This is also consistent with our findings that high *SOX9* expression was associated with an unfavorable prognosis in CC.

However, *SOX9* has shown the opposite effect in some studies. Wu et al. (2013) screened hypermethylated genes in 48 normal cervices, 30 CIN I, 30 CIN II-III, and 48 cervical squamous cell carcinomas, for a total of 156 samples. They found that the methylation level of *SOX9* increased with tumor severity. Their study was based on the view that *SOX9* is a tumor suppressor gene because CpG islands methylated in promoter regions can silence transcription (Hou et al., 2021). Wang et al. (2015b) found that overexpression of *SOX9* could inhibit the tumor formation, proliferation, and viability of CC cells *in vitro*; the protein expression of *SOX9* gradually decreased in normal cervical tissues, CIN III, and cervical cancer tissues. Therefore, *SOX9* has thus been proposed as a possible cancer-inhibiting gene for CC. Thus, more studies are needed to prove its role in CC. It was interesting to us that the long control region **(**LCR) of HPV16 and HPV52 was found to have a high permanent binding site for the *SOX9* transcription factor (Xi et al., 2017; Song et al., 2021; Mane et al., 2022), and these may be new targets for the treatment of HPV-associated CC.

*SOX9* also plays an important role in other types of gynecological malignancies. In ovarian epithelial tumors, many studies have found that *SOX9* is significantly higher in tumor tissue than in normal ovarian tissue, and its expression correlates with overall survival and progression-free survival of ovarian cancer patients; elevated expression of some miRNAs can suppress *SOX9* expression, thereby inhibiting ovarian cancer cell growth and multicellular spheroids formation; ceRNA network-regulated *SOX9* expression is involved in the development of ovarian cancer, maintenance of tumor stem cell properties, anti-apoptosis, migration, and invasion; meanwhile, *SOX9* expression is associated with chemoresistance resistance in ovarian cancer patients (Chen et al., 2021). Recent studies have also demonstrated that cytoplasmic *SOX9*-mediated cell death inhibition contributes to the survival of cancer stem cells in high-grade ovarian cancer (Oh et al., 2022). In endometrial cancer, upregulation of *SOX9* could promote endometrial cell proliferation, induce endometrial precancerous lesions, and predict the prognosis of endometrial cancer patients in combination with the expression of other genes. In uterine sarcoma, upregulation of *SOX9* promotes epithelial-mesenchymal transition, leading to sarcoma formation (Chen et al., 2021).

Emerging evidence also suggests that *SOX9* interacts with the tumor immune microenvironment (Panda et al., 2021). In cancers, activated fibroblasts, which are called CAF, make up a major component of the tumor microenvironment (Zu et al., 2020). It is composed of about 40%–50% of the total cell population of cancer patients (Wang et al., 2017c) and plays a major role in cancer development. Recent studies also suggest a role for CAFs in regulating *SOX9* expression in prostate cancer (Qin et al., 2021). Thus, the interaction of CAF with SOX9 may be important in the development of certain cancers.

## Conclusion

According to our findings, *SOX9* expression level was significantly related to the OS and PFS of CC. Compared to cancer adjacent normal tissues, both mRNA and the protein expression of *SOX9* in CC tissue were higher. The expression of *SOX9* correlates with cancer-associated fibroblast immune infiltration in immune cells. Furthermore, cancer-associated fibroblast infiltration is associated with CC patients’ prognosis. Therefore, the *SOX9* gene acts as an oncogene in CC and interacts with immune infiltration of cancer-associated fibroblasts, thereby affecting the prognosis of patients with cervical cancer.

## Data Availability

The datasets presented in this study can be found in online repositories. The names of the repository/repositories and accession number(s) can be found in the article/Supplementary Material.

## References

[B1] Ashrafizadeh M. Zarrabi A. Orouei S. Zabolian A. Saleki H. Azami N. (2021). Interplay between SOX9 transcription factor and microRNAs in cancer. Int. J. Biol. Macromol. 183, 681–694. Epub 2021 May 3. PMID: 33957202. 10.1016/j.ijbiomac.2021.04.185 PubMed Abstract | 10.1016/j.ijbiomac.2021.04.185 | Google Scholar 33957202

[B2] Bengtsson H. Ray A. Spellman P. Speed T. P. (2009). A single-sample method for normalizing and combining full-resolution copy numbers from multiple platforms, labs and analysis methods. Bioinformatics 25 (7), 861–867. 10.1093/bioinformatics/btp074 PubMed Abstract | 10.1093/bioinformatics/btp074 | Google Scholar 19193730 PMC2660872

[B3] Burnichon N. Buffet A. Parfait B. Letouze E. Laurendeau I. Loriot C. (2012). Somatic NF1 inactivation is a frequent event in sporadic pheochromocytoma. Hum. Mol. Genet. 21 (26), 5397–5405. 10.1093/hmg/dds374 PubMed Abstract | 10.1093/hmg/dds374 | Google Scholar 22962301

[B4] Cai C. Wang H. He H. H. Chen S. He L. Ma F. (2013). ERG induces androgen receptor-mediated regulation of SOX9 in prostate cancer. J. Clin. Invest. 123 (3), 1109–1122. 10.1172/JCI66666 PubMed Abstract | 10.1172/JCI66666 | Google Scholar 23426182 PMC3582143

[B5] Chang C. V. Araujo R. V. Cirqueira C. S. Cani C. M. Matushita H. Cescato V. A. (2017). Differential expression of stem cell markers in human adamantinomatous craniopharyngioma and pituitary adenoma. Neuroendocrinology 104 (2), 183–193. 10.1159/000446072 PubMed Abstract | 10.1159/000446072 | Google Scholar 27161333

[B6] Che L. F. Shao S. F. Wang L. X. (2016). Downregulation of CCR5 inhibits the proliferation and invasion of cervical cancer cells and is regulated by microRNA-107. Exp. Ther. Med. 11 (2), 503–509. 10.3892/etm.2015.2911 PubMed Abstract | 10.3892/etm.2015.2911 | Google Scholar 26893637 PMC4734227

[B7] Chen H. He Y. Wen X. Shao S. Liu Y. Wang J. (2021). SOX9: Advances in gynecological malignancies. Front. Oncol. 11, 768264. PMID: 34881182PMCIDPMC5360585. 10.3389/fonc.2021.768264 PubMed Abstract | 10.3389/fonc.2021.768264 | Google Scholar 34881182 PMC8645898

[B8] Chen X. Wei S. Ma H. Jin G. Hu Z. Suping H. (2019). Telomere length in cervical exfoliated cells, interaction with HPV genotype, and cervical cancer occurrence among high-risk HPV-positive women. Cancer Med. 8 (10), 4845–4851. 10.1002/cam4.2246 PubMed Abstract | 10.1002/cam4.2246 | Google Scholar 31243901 PMC6712472

[B9] Chen X. Xiong D. Ye L. Wang K. Huang L. Mei S. (2019). Up-regulated lncRNA XIST contributes to progression of cervical cancer via regulating miR-140-5p and ORC1. Cancer Cell Int. 19, 45. 10.1186/s12935-019-0744-y PubMed Abstract | 10.1186/s12935-019-0744-y | Google Scholar 30858762 PMC6394057

[B10] Chen X. Xu H. Xu W. Zeng W. Liu J. Wu Q. (2017). Prevalence and genotype distribution of human papillomavirus in 961, 029 screening tests in southeastern China (Zhejiang Province) between 2011 and 2015. Sci. Rep. 7 (1), 14813. 10.1038/s41598-017-13299-y PubMed Abstract | 10.1038/s41598-017-13299-y | Google Scholar 29093458 PMC5665931

[B11] de Bont J. M. Kros J. M. Passier M. M. Reddingius R. E. Sillevis Smitt P. A. Luider T. M. (2008). Differential expression and prognostic significance of SOX genes in pediatric medulloblastoma and ependymoma identified by microarray analysis. Neuro. Oncol. 10 (5), 648–660. 10.1215/15228517-2008-032 PubMed Abstract | 10.1215/15228517-2008-032 | Google Scholar 18577562 PMC2666242

[B12] Drivdahl R. Haugk K. H. Sprenger C. C. Nelson P. S. Tennant M. K. Plymate S. R. (2004). Suppression of growth and tumorigenicity in the prostate tumor cell line M12 by overexpression of the transcription factor SOX9. Oncogene 23 (26), 4584–4593. 10.1038/sj.onc.1207603 PubMed Abstract | 10.1038/sj.onc.1207603 | Google Scholar 15077158

[B13] Feng C. Ma F. Hu C. Ma J. A. Wang J. Zhang Y. (2018). SOX9/miR-130a/CTR1 axis modulates DDP-resistance of cervical cancer cell. Cell Cycle 17 (4), 448–458. 10.1080/15384101.2017.1395533 PubMed Abstract | 10.1080/15384101.2017.1395533 | Google Scholar 29099271 PMC5927693

[B14] Galmiche L. Sarnacki S. Verkarre V. Boizet B. Duvillie B. Fabre M. (2008). Transcription factors involved in pancreas development are expressed in paediatric solid pseudopapillary tumours. Histopathology 53 (3), 318–324. 10.1111/j.1365-2559.2008.03108.x PubMed Abstract | 10.1111/j.1365-2559.2008.03108.x | Google Scholar 18671802

[B15] Gao M. Kong W. Huang Z. Xie Z. (2020). Identification of key genes related to lung squamous cell carcinoma using bioinformatics analysis. Int. J. Mol. Sci. 21 (8), 2994. 10.3390/ijms21082994 10.3390/ijms21082994 | Google Scholar PMC7215920 32340320

[B16] Gong L. Ji H. H. Tang X. W. Pan L. Y. Chen X. Jia Y. T. (2020). Human papillomavirus vaccine-associated premature ovarian insufficiency and related adverse events: data mining of vaccine adverse event reporting system. Sci. Rep. 10 (1), 10762. 10.1038/s41598-020-67668-1 PubMed Abstract | 10.1038/s41598-020-67668-1 | Google Scholar 32612121 PMC7329819

[B17] Guo X. Xiong L. Sun T. Peng R. Zou L. Zhu H. (2012). Expression features of SOX9 associate with tumor progression and poor prognosis of hepatocellular carcinoma. Diagn. Pathol. 7, 44. 10.1186/1746-1596-7-44 PubMed Abstract | 10.1186/1746-1596-7-44 | Google Scholar 22515642 PMC3464896

[B18] He S. Feng Y. Zou W. Wang J. Li G. Xiong W. (2022). The role of the SOX9/lncRNA ANXA2P2/miR-361-3p/SOX9 regulatory loop in cervical cancer cell growth and resistance to cisplatin. Front. Oncol. 11, 784525. PMID: 35083143; PMCID: PMC8784813. 10.3389/fonc.2021.784525 PubMed Abstract | 10.3389/fonc.2021.784525 | Google Scholar 35083143 PMC8784813

[B19] Hou Y. Hu J. Zhou L. Liu L. Chen K. Yang X. (2021). Integrative analysis of methylation and copy number variations of prostate adenocarcinoma based on weighted gene Co-expression network analysis. Front. Oncol. 11, 647253. 10.3389/fonc.2021.647253 PubMed Abstract | 10.3389/fonc.2021.647253 | Google Scholar 33869043 PMC8047072

[B20] Huang da W. Sherman B. T. Lempicki R. A. (2009). Bioinformatics enrichment tools: paths toward the comprehensive functional analysis of large gene lists. Nucleic Acids Res. 37 (1), 1–13. 10.1093/nar/gkn923 PubMed Abstract | 10.1093/nar/gkn923 | Google Scholar 19033363 PMC2615629

[B21] Huang da W. Sherman B. T. Lempicki R. A. (2009). Systematic and integrative analysis of large gene lists using DAVID bioinformatics resources. Nat. Protoc. 4 (1), 44–57. 10.1038/nprot.2008.211 PubMed Abstract | 10.1038/nprot.2008.211 | Google Scholar 19131956

[B22] Huang J. Q. Wei F. K. Xu X. L. Ye S. X. Song J. W. Ding P. K. (2019). SOX9 drives the epithelial-mesenchymal transition in non-small-cell lung cancer through the Wnt/β-catenin pathway. J. Transl. Med. 17 (1), 143. 10.1186/s12967-019-1895-2 PubMed Abstract | 10.1186/s12967-019-1895-2 | Google Scholar 31060551 PMC6501400

[B23] Jiang S. S. Fang W. T. Hou Y. H. Huang S. F. Yen B. L. Chang J. L. (2010). Upregulation of SOX9 in lung adenocarcinoma and its involvement in the regulation of cell growth and tumorigenicity. Clin. Cancer Res. 16 (17), 4363–4373. 10.1158/1078-0432.CCR-10-0138 PubMed Abstract | 10.1158/1078-0432.CCR-10-0138 | Google Scholar 20651055

[B24] Jin Y. Zhou X. Yao X. Zhang Z. Cui M. Lin Y. (2020). MicroRNA-612 inhibits cervical cancer progression by targeting NOB1. J. Cell. Mol. Med. 24 (5), 3149–3156. 10.1111/jcmm.14985 PubMed Abstract | 10.1111/jcmm.14985 | Google Scholar 31970934 PMC7077537

[B25] Kim A. L. Back J. H. Chaudhary S. C. Zhu Y. Athar M. Bickers D. R. (2018). SOX9 transcriptionally regulates mTOR-induced proliferation of basal cell carcinomas. J. Invest. Dermatol. 138 (8), 1716–1725. 10.1016/j.jid.2018.01.040 PubMed Abstract | 10.1016/j.jid.2018.01.040 | Google Scholar 29550418 PMC6056318

[B26] Langfelder P. Horvath S. (2008). Wgcna: an R package for weighted correlation network analysis. BMC Bioinforma. 9, 559. 10.1186/1471-2105-9-559 10.1186/1471-2105-9-559 | Google Scholar PMC2631488 19114008

[B27] Langfelder Peter Horvath Steve (2012). Fast R functions for robust correlations and hierarchical clustering. J. Stat. Softw. 46 (11), 1–17. 10.18637/jss.v046.i11 PubMed Abstract | 10.18637/jss.v046.i11 | Google Scholar 23050260 PMC3465711

[B28] Lei Z. Shi H. Li W. Yu D. Shen F. Yu X. (2018). miR-185 inhibits non-small cell lung cancer cell proliferation and invasion through targeting of SOX9 and regulation of Wnt signaling. Mol. Med. Rep. 17 (1), 1742–1752. 10.3892/mmr.2017.8050 PubMed Abstract | 10.3892/mmr.2017.8050 | Google Scholar 29138830 PMC5780119

[B29] Li C. Ao H. Chen G. Wang F. Li F. (2020). The interaction of CDH20 with β-catenin inhibits cervical cancer cell migration and invasion via TGF-β/smad/SNAIL mediated EMT. Front. Oncol. 9, 1481. 10.3389/fonc.2019.01481 PubMed Abstract | 10.3389/fonc.2019.01481 | Google Scholar 31998642 PMC6962355

[B30] Li L. Lv J. He Y. Wang Z. (2020). Gene network in pulmonary tuberculosis based on bioinformatic analysis. BMC Infect. Dis. 20 (1), 612. 10.1186/s12879-020-05335-6 PubMed Abstract | 10.1186/s12879-020-05335-6 | Google Scholar 32811479 PMC7436983

[B31] Li M. Jin X. Li H. Wu G. Wang S. Yang C. (2020). Key genes with prognostic values in suppression of osteosarcoma metastasis using comprehensive analysis. BMC cancer 20 (1), 65. 10.1186/s12885-020-6542-z PubMed Abstract | 10.1186/s12885-020-6542-z | Google Scholar 31992246 PMC6988291

[B32] Li Q. Wang Q. Zhang Q. Zhang J. Zhang J. (2019). Collagen prolyl 4-hydroxylase 2 predicts worse prognosis and promotes glycolysis in cervical cancer. Am. J. Transl. Res. 11 (11), 6938–6951. PubMed Abstract | Google Scholar 31814898 PMC6895525

[B33] Li T. Fu J. Zeng Z. Cohen D. Li J. Chen Q. (2020). TIMER2.0 for analysis of tumor-infiltrating immune cells. Nucleic Acids Res. 48 (W1), W509–W514. PMCIDPMC5360585. 10.1093/nar/gkaa407 PubMed Abstract | 10.1093/nar/gkaa407 | Google Scholar 32442275 PMC7319575

[B34] Li W. Tian S. Wang P. Zang Y. Chen X. Yao Y. (2019). The characteristics of HPV integration in cervical intraepithelial cells. J. Cancer 10 (12), 2783–2787. 10.7150/jca.31450 PubMed Abstract | 10.7150/jca.31450 | Google Scholar 31258786 PMC6584928

[B35] Li X. M. Piao Y. J. Sohn K. C. Ha J. M. Im M. Seo Y. J. (2016). Sox9 is a β-catenin-regulated transcription factor that enhances the colony-forming activity of squamous cell carcinoma cells. Mol. Med. Rep. 14 (1), 337–342. PMID: 27151141. 10.3892/mmr.2016.5210 PubMed Abstract | 10.3892/mmr.2016.5210 | Google Scholar 27151141

[B36] Lin F. Xie Y. J. Zhang X. K. Huang T. J. Xu H. F. Mei Y. (2019). GTSE1 is involved in breast cancer progression in p53 mutation-dependent manner. J. Exp. Clin. Cancer Res. 38 (1), 152. 10.1186/s13046-019-1157-4 PubMed Abstract | 10.1186/s13046-019-1157-4 | Google Scholar 30961661 PMC6454633

[B37] Lin M. Ye M. Zhou J. Wang Z. P. Zhu X. (2019). Recent advances on the molecular mechanism of cervical carcinogenesis based on systems biology technologies. Comput. Struct. Biotechnol. J. 17, 241–250. Published 2019 Feb 7. 10.1016/j.csbj.2019.02.001 PubMed Abstract | 10.1016/j.csbj.2019.02.001 | Google Scholar 30847042 PMC6389684

[B38] Liu C. Q. Chen Y. Xie B. F. Li Y. L. Wei Y. T. Wang F. (2019). MicroRNA-215-3p suppresses the growth and metastasis of cervical cancer cell via targeting SOX9. Eur. Rev. Med. Pharmacol. Sci. 23 (13), 5628–5639. 10.26355/eurrev_201907_18297 PubMed Abstract | 10.26355/eurrev_201907_18297 | Google Scholar 31298315

[B39] Liu C. Ren Y. F. Dong J. Ke M. Y. Ma F. Monga S. (2017). Activation of SRY accounts for male-specific hepatocarcinogenesis: Implication in gender disparity of hepatocellular carcinoma. Cancer Lett. 410, 20–31. 10.1016/j.canlet.2017.09.013 PubMed Abstract | 10.1016/j.canlet.2017.09.013 | Google Scholar 28942012

[B40] Liu Y. Song Y. Hu X. Yan L. Zhu X. (2019). Awareness of surgical smoke hazards and enhancement of surgical smoke prevention among the gynecologists. J. Cancer 10 (12), 2788–2799. Published 2019 Jun 2. 10.7150/jca.31464 PubMed Abstract | 10.7150/jca.31464 | Google Scholar 31258787 PMC6584931

[B41] Liu Y. Tingart M. Lecouturier S. Li J. Eschweiler J. (2021). Identification of co-expression network correlated with different periods of adipogenic and osteogenic differentiation of BMSCs by weighted gene co-expression network analysis (WGCNA). BMC Genomics 22 (1), 254. 10.1186/s12864-021-07584-4 PubMed Abstract | 10.1186/s12864-021-07584-4 | Google Scholar 33836657 PMC8035768

[B42] Ma Y. Shepherd J. Zhao D. Bollu L. R. Tahaney W. M. Hill J. (2020). SOX9 is essential for triple-negative breast cancer cell survival and metastasis. Mol. Cancer Res. 18 (12), 1825–1838. 10.1158/1541-7786.MCR-19-0311 PubMed Abstract | 10.1158/1541-7786.MCR-19-0311 | Google Scholar 32661114 PMC7718423

[B43] Mane A. Limaye S. Patil L. Kulkarni-Kale U. (2022). Genetic variations in the long control region of human papillomavirus type 16 isolates from India: implications for cervical carcinogenesis. J. Med. Microbiol. 71 (1). 10.1099/jmm.0.001475 10.1099/jmm.0.001475 | Google Scholar 35040427

[B44] Matheu A. Collado M. Wise C. Manterola L. Cekaite L. Tye A. J. (2012). Oncogenicity of the developmental transcription factor Sox9. Cancer Res. 72 (5), 1301–1315. 10.1158/0008-5472.CAN-11-3660 PubMed Abstract | 10.1158/0008-5472.CAN-11-3660 | Google Scholar 22246670 PMC3378515

[B45] Matsushima H. Kuroki T. Kitasato A. Adachi T. Tanaka T. Hirabaru M. (2015). Sox9 expression in carcinogenesis and its clinical significance in intrahepatic cholangiocarcinoma. Dig. Liver Dis. 47 (12), 1067–1075. 10.1016/j.dld.2015.08.003 PubMed Abstract | 10.1016/j.dld.2015.08.003 | Google Scholar 26341967

[B46] Oh M. Son C. Rho S. B. Kim M. Park K. Song S. Y. (2022). Stem cell factor SOX9 interacts with a cell death regulator RIPK1 and results in escape of cancer stem cell death. Cells 11 (3), 363. PMCIDPMC5360585. 10.3390/cells11030363 PubMed Abstract | 10.3390/cells11030363 | Google Scholar 35159173 PMC8834197

[B47] Panda M. Tripathi S. K. Biswal B. K. (2021). SOX9: An emerging driving factor from cancer progression to drug resistance. Biochim. Biophys. Acta. Rev. Cancer 1875 (2), 188517. Epub 2021 Jan 29. PMID: 33524528. 10.1016/j.bbcan.2021.188517 PubMed Abstract | 10.1016/j.bbcan.2021.188517 | Google Scholar 33524528

[B48] Paskeh M. D. A. Mirzaei S. Gholami M. H. Zarrabi A. Zabolian A. Hashemi M. (2021). Cervical cancer progression is regulated by SOX transcription factors: Revealing signaling networks and therapeutic strategies. Biomed. Pharmacother. 144, 112335. PMID: 34700233. 10.1016/j.biopha.2021.112335 PubMed Abstract | 10.1016/j.biopha.2021.112335 | Google Scholar 34700233

[B49] Paz Soldan V. A. Lee F. H. Carcamo C. Holmes K. K. Garnett G. P. Garcia P. (2008). Who is getting Pap smears in urban Peru? Int. J. Epidemiol. 37 (4), 862–869. 10.1093/ije/dyn118 PubMed Abstract | 10.1093/ije/dyn118 | Google Scholar 18653515 PMC2734064

[B50] Pomp V. Leo C. Mauracher A. Korol D. Guo W. Varga Z. (2015). Differential expression of epithelial–mesenchymal transition and stem cell markers in intrinsic subtypes of breast cancer. Breast Cancer Res. Treat. 154 (1), 45–55. 10.1007/s10549-015-3598-6 PubMed Abstract | 10.1007/s10549-015-3598-6 | Google Scholar 26467042

[B51] Qin H. Yang Y. Jiang B. Pan C. Chen W. Diao W. (2021). SOX9 in prostate cancer is upregulated by cancer-associated fibroblasts to promote tumor progression through HGF/c-Met-FRA1 signaling. FEBS J. 288 (18), 5406–5429. Epub 2021 Apr 2. PMID: 33705609. 10.1111/febs.15816 PubMed Abstract | 10.1111/febs.15816 | Google Scholar 33705609

[B52] Ritchie M. E. Phipson B. Wu D. Hu Y. Law C. W. Shi W. (2015). limma powers differential expression analyses for RNA-sequencing and microarray studies. Nucleic Acids Res. 43 (7), e47. 10.1093/nar/gkv007 PubMed Abstract | 10.1093/nar/gkv007 | Google Scholar 25605792 PMC4402510

[B53] Sakamoto H. Mutoh H. Miura Y. Sashikawa M. Yamamoto H. Sugano K. (2013). SOX9 is highly expressed in nonampullary duodenal adenoma and adenocarcinoma in humans. Gut Liver 7 (5), 513–518. 10.5009/gnl.2013.7.5.513 PubMed Abstract | 10.5009/gnl.2013.7.5.513 | Google Scholar 24073307 PMC3782664

[B54] Shannon P. Markiel A. Ozier O. Baliga N. S. Wang J. T. Ramage D. (2003). Cytoscape: a software environment for integrated models of biomolecular interaction networks. Genome Res. 13 (11), 2498–2504. 10.1101/gr.1239303 PubMed Abstract | 10.1101/gr.1239303 | Google Scholar 14597658 PMC403769

[B55] Shefchek K. A. Harris N. L. Gargano M. Matentzoglu N. Unni D. Brush M. (2020). The monarch initiative in 2019: an integrative data and analytic platform connecting phenotypes to genotypes across species. Nucleic Acids Res. 48 (D1), D704–D715. 10.1093/nar/gkz997 PubMed Abstract | 10.1093/nar/gkz997 | Google Scholar 31701156 PMC7056945

[B56] Song Z. Cui Y. Li Q. Deng J. Ding X. He J. (20212021). The genetic variability, phylogeny and functional significance of E6, E7 and LCR in human papillomavirus type 52 isolates in Sichuan, China. Virol. J. 18 (1), 94. 10.1186/s12985-021-01565-5 10.1186/s12985-021-01565-5 | Google Scholar PMC8091156 33941222

[B57] Tang Z. Kang B. Li C. Chen T. Zhang Z. (2019). GEPIA2: an enhanced web server for large-scale expression profiling and interactive analysis. Nucleic Acids Res. 47 (W1), W556–W560. 10.1093/nar/gkz430 PubMed Abstract | 10.1093/nar/gkz430 | Google Scholar 31114875 PMC6602440

[B58] Tremblay B. L. Guénard F. Lamarche B. Pérusse L. Vohl M. C. (2019). Network analysis of the potential role of DNA methylation in the relationship between plasma carotenoids and lipid profile. Nutrients 11 (6), 1265. 10.3390/nu11061265 10.3390/nu11061265 | Google Scholar PMC6628241 31167428

[B59] Tripathi S. K. Sahoo R. K. Biswal B. K. (2022). SOX9 as an emerging target for anticancer drugs and a prognostic biomarker for cancer drug resistance. Drug Discov. Today 27, S11359-6446(22)00213-6. Epub ahead of print. PMID: 35636723. 10.1016/j.drudis.2022.05.022 10.1016/j.drudis.2022.05.022 | Google Scholar 35636723

[B60] Tsuda M. Fukuda A. Roy N. Hiramatsu Y. Leonhardt L. Kakiuchi N. (2018). The BRG1/SOX9 axis is critical for acinar cell-derived pancreatic tumorigenesis. J. Clin. Invest. 128 (8), 3475–3489. 10.1172/JCI94287 PubMed Abstract | 10.1172/JCI94287 | Google Scholar 30010625 PMC6063489

[B61] Uhlen M. Zhang C. Lee S. Sjöstedt E. Fagerberg L. Bidkhori G. (2017). A pathology atlas of the human cancer transcriptome. Sci. (New York, N.Y.) 357 (6352), eaan2507. 10.1126/science.aan2507 10.1126/science.aan2507 | Google Scholar 28818916

[B62] Voronkova M. A. Rojanasakul L. W. Kiratipaiboon C. Rojanasakul Y. (2020). The SOX9-aldehyde dehydrogenase Axis determines resistance to chemotherapy in non-small-cell lung cancer. Mol. Cell. Biol. 40 (2), e00307-19. Published 2020 Jan 3. 10.1128/MCB.00307-19 PubMed Abstract | 10.1128/MCB.00307-19 | Google Scholar 31658996 PMC6944474

[B63] Wan Y. P. Xi M. He H. C. Wan S. Hua W. Zen Z. C. (2017). Expression and clinical significance of SOX9 in renal cell carcinoma, bladder cancer and penile cancer. Oncol. Res. Treat. 40 (1-2), 15–20. 10.1159/000455145 PubMed Abstract | 10.1159/000455145 | Google Scholar 28118628

[B64] Wang H. Tang M. Ou L. Hou M. Feng T. Huang Y. E. (2017). Biological analysis of cancer specific microRNAs on function modeling in osteosarcoma. Sci. Rep. 7 (1), 5382. 10.1038/s41598-017-05819-7 PubMed Abstract | 10.1038/s41598-017-05819-7 | Google Scholar 28710380 PMC5511279

[B65] Wang H. Y. Lian P. Zheng P. S. (2015). SOX9, a potential tumor suppressor in cervical cancer, transactivates p21WAF1/CIP1 and suppresses cervical tumor growth. Oncotarget 6 (24), 20711–20722. 10.18632/oncotarget.4133 PubMed Abstract | 10.18632/oncotarget.4133 | Google Scholar 26036262 PMC4653037

[B66] Wang L. He S. Yuan J. Mao X. Cao Y. Zong J. (2012). Oncogenic role of SOX9 expression in human malignant glioma. Med. Oncol. 29 (5), 3484–3490. 10.1007/s12032-012-0267-z PubMed Abstract | 10.1007/s12032-012-0267-z | Google Scholar 22714060

[B67] Wang L. Yu X. Zhang Z. Pang L. Xu J. Jiang J. (2017). Linc-ROR promotes esophageal squamous cell carcinoma progression through the derepression of SOX9. J. Exp. Clin. Cancer Res. 36 (1), 182. 10.1186/s13046-017-0658-2 PubMed Abstract | 10.1186/s13046-017-0658-2 | Google Scholar 29237490 PMC5727696

[B68] Wang W. T. Qi Q. Zhao P. Li C. Y. Yin X. Y. Yan R. B. (2018). miR-590-3p is a novel microRNA which suppresses osteosarcoma progression by targeting SOX9. Biomed. Pharmacother. 107, 1763–1769. 10.1016/j.biopha.2018.06.124 PubMed Abstract | 10.1016/j.biopha.2018.06.124 | Google Scholar 30257395

[B69] Wang X. Ju Y. Zhou M. I. Liu X. Zhou C. (2015). Upregulation of SOX9 promotes cell proliferation, migration and invasion in lung adenocarcinoma. Oncol. Lett. 10 (2), 990–994. 10.3892/ol.2015.3303 PubMed Abstract | 10.3892/ol.2015.3303 | Google Scholar 26622611 PMC4509053

[B70] Wang Y. Gan G. Wang B. Wu J. Cao Y. Zhu D. (2017). Cancer-associated fibroblasts promote irradiated cancer cell recovery through autophagy. EBioMedicine 17, 45–56. Epub 2017 Feb 22. PMID: 28258923PMCIDPMC5360585. 10.1016/j.ebiom.2017.02.019 PubMed Abstract | 10.1016/j.ebiom.2017.02.019 | Google Scholar 28258923 PMC5360585

[B71] Wu J. H. Liang X. A. Wu Y. M. Li F. S. Dai Y. M. (2013). Identification of DNA methylation of SOX9 in cervical cancer using methylated-CpG island recovery assay. Oncol. Rep. 29 (1), 125–132. 10.3892/or.2012.2077 PubMed Abstract | 10.3892/or.2012.2077 | Google Scholar 23064448

[B72] Xi J. Chen J. Xu M. Yang H. Luo J. Pan Y. (2017). Genetic variability and functional implication of the long control region in HPV-16 variants in Southwest China. PLoS One 12 (8), e0182388. 10.1371/journal.pone.0182388 PubMed Abstract | 10.1371/journal.pone.0182388 | Google Scholar 28767682 PMC5540483

[B73] Xing S. Wang Y. Hu K. Wang F. Sun T. Li Q. (2020). WGCNA reveals key gene modules regulated by the combined treatment of colon cancer with PHY906 and CPT11. Biosci. Rep. 40 (9), BSR20200935. 10.1042/BSR20200935 PubMed Abstract | 10.1042/BSR20200935 | Google Scholar 32812032 PMC7468096

[B74] Xu Z. Wu Z. Xu J. Zhang J. Yu B. (2020). Identification of hub driving genes and regulators of lung adenocarcinoma based on the gene Co-expression network. Biosci. Rep. 40 (4), BSR20200295. 10.1042/BSR20200295 PubMed Abstract | 10.1042/BSR20200295 | Google Scholar 32196072 PMC7108999

[B75] Yamashita S. Kataoka K. Yamamoto H. Kato T. Hara S. Yamaguchi K. (2019). Comparative analysis demonstrates cell type-specific conservation of SOX9 targets between mouse and chicken. Sci. Rep. 9 (1), 12560. 10.1038/s41598-019-48979-4 PubMed Abstract | 10.1038/s41598-019-48979-4 | Google Scholar 31467356 PMC6715657

[B76] Yang X. Liang R. Liu C. Liu J. A. Cheung M. Liu X. (2019). SOX9 is a dose-dependent metastatic fate determinant in melanoma. J. Exp. Clin. Cancer Res. 38 (1), 17. 10.1186/s13046-018-0998-6 PubMed Abstract | 10.1186/s13046-018-0998-6 | Google Scholar 30642390 PMC6330758

[B77] Ye J. Jin C. F. Li N. Liu M. H. Fei Z. X. Dong L. Z. (2018). Selection of suitable reference genes for qRT-PCR normalisation under different experimental conditions in Eucommia ulmoides Oliv. Sci. Rep. 8 (1), 15043. 10.1038/s41598-018-33342-w PubMed Abstract | 10.1038/s41598-018-33342-w | Google Scholar 30301911 PMC6177395

[B78] Yu C. C. Tsai L. L. Wang M. L. Yu C. H. Lo W. L. Chang Y. C. (2013). miR145 targets the SOX9/ADAM17 axis to inhibit tumor-initiating cells and IL-6-mediated paracrine effects in head and neck cancer. Cancer Res. 73 (11), 3425–3440. 10.1158/0008-5472.CAN-12-3840 PubMed Abstract | 10.1158/0008-5472.CAN-12-3840 | Google Scholar 23548270

[B79] Zhang J. Tong Y. Ren L. Li C. D. (2014). Expression of metastasis suppressor 1 in cervical carcinoma and the clinical significance. Oncol. Lett. 8 (5), 2145–2149. 10.3892/ol.2014.2508 PubMed Abstract | 10.3892/ol.2014.2508 | Google Scholar 25295101 PMC4186592

[B80] Zhang X. D. Wang Y. N. Feng X. Y. Yang J. Y. Ge Y. Y. Kong W. Q. (2018). Biological function of microRNA-30c/SOX9 in pediatric osteosarcoma cell growth and metastasis. Eur. Rev. Med. Pharmacol. Sci. 22 (1), 70–78. 10.26355/eurrev_201801_14102 PubMed Abstract | 10.26355/eurrev_201801_14102 | Google Scholar 29364496

[B81] Zhao J. Chang L. Gu X. Liu J. Sun B. Wei X. (2020). Systematic profiling of alternative splicing signature reveals prognostic predictor for prostate cancer. Cancer Sci. 111 (8), 3020–3031. 10.1111/cas.14525 PubMed Abstract | 10.1111/cas.14525 | Google Scholar 32530556 PMC7419053

[B82] Zhao J. Li H. Yuan M. (2021). EGR1 promotes stemness and predicts a poor outcome of uterine cervical cancer by inducing SOX9 expression. Genes Genomics 43 (5), 459–470. 10.1007/s13258-021-01064-5 PubMed Abstract | 10.1007/s13258-021-01064-5 | Google Scholar 33687657

[B83] Zhou C. H. Ye L. P. Ye S. X. Li Y. Zhang X. Y. Xu X. Y. (2012). Clinical significance of SOX9 in human non-small cell lung cancer progression and overall patient survival. J. Exp. Clin. Cancer Res. 31 (1), 18. 10.1186/1756-9966-31-18 PubMed Abstract | 10.1186/1756-9966-31-18 | Google Scholar 22385677 PMC3313873

[B84] Zhou C. J. Guo J. Q. Zhu K. X. Zhang Q. H. Pan C. R. Xu W. H. (2011). Elevated expression of SOX9 is related with the progression of gastric carcinoma. Diagn. Cytopathol. 39 (2), 105–109. 10.1002/dc.21348 PubMed Abstract | 10.1002/dc.21348 | Google Scholar 20301211

[B85] Zu T. Wen J. Xu L. Li H. Mi J. Li H. (2020). Up-regulation of activating transcription factor 3 in human fibroblasts inhibits melanoma cell growth and migration through a paracrine pathway. Front. Oncol. 10, 624. PMID: 32373541PMCIDPMC5360585. 10.3389/fonc.2020.00624 PubMed Abstract | 10.3389/fonc.2020.00624 | Google Scholar 32373541 PMC7187895

