# Supplementary figures and images for: Prognostic value of SOX9 in cervical cancer: Bioinformatics and experimental approaches
Source: Front Genet. 2022 Aug 8;13:939328. doi: 10.3389/fgene.2022.939328 (PMC9394184; doi:10.3389/fgene.2022.939328)

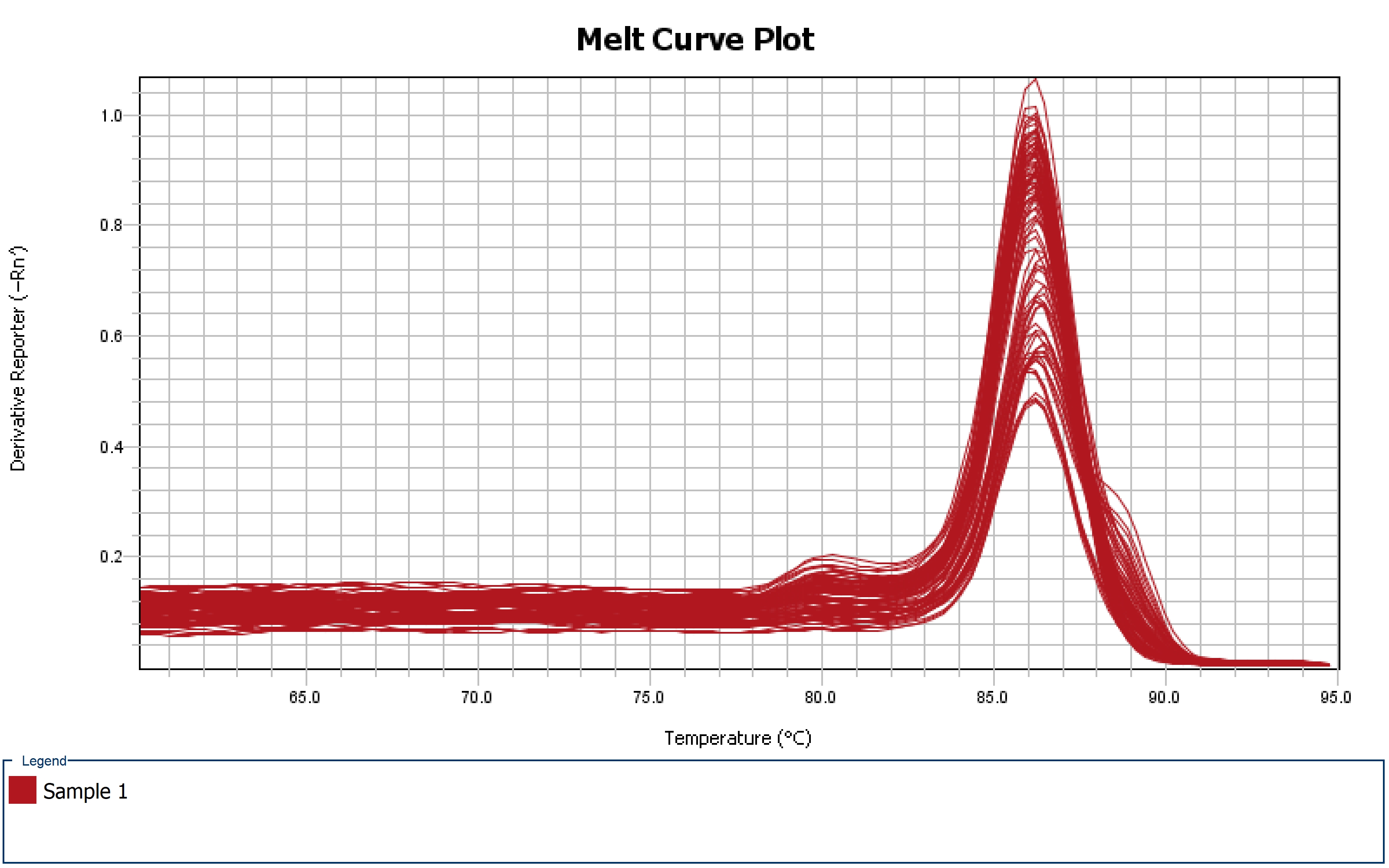

Supplement: Supplementary file 1 [file Image3.JPEG]

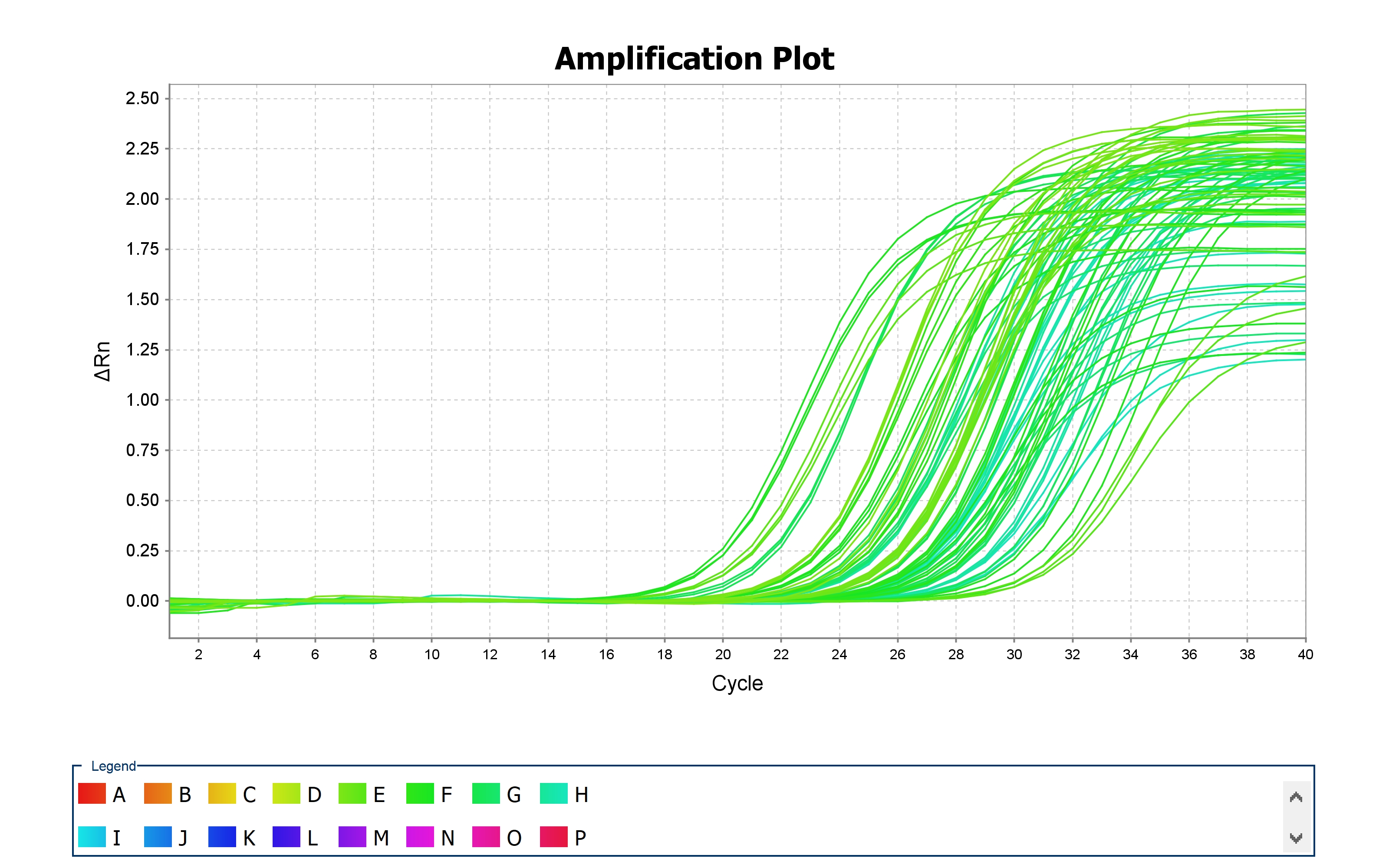

Supplement: Supplementary file 2 [file Image1.JPEG]

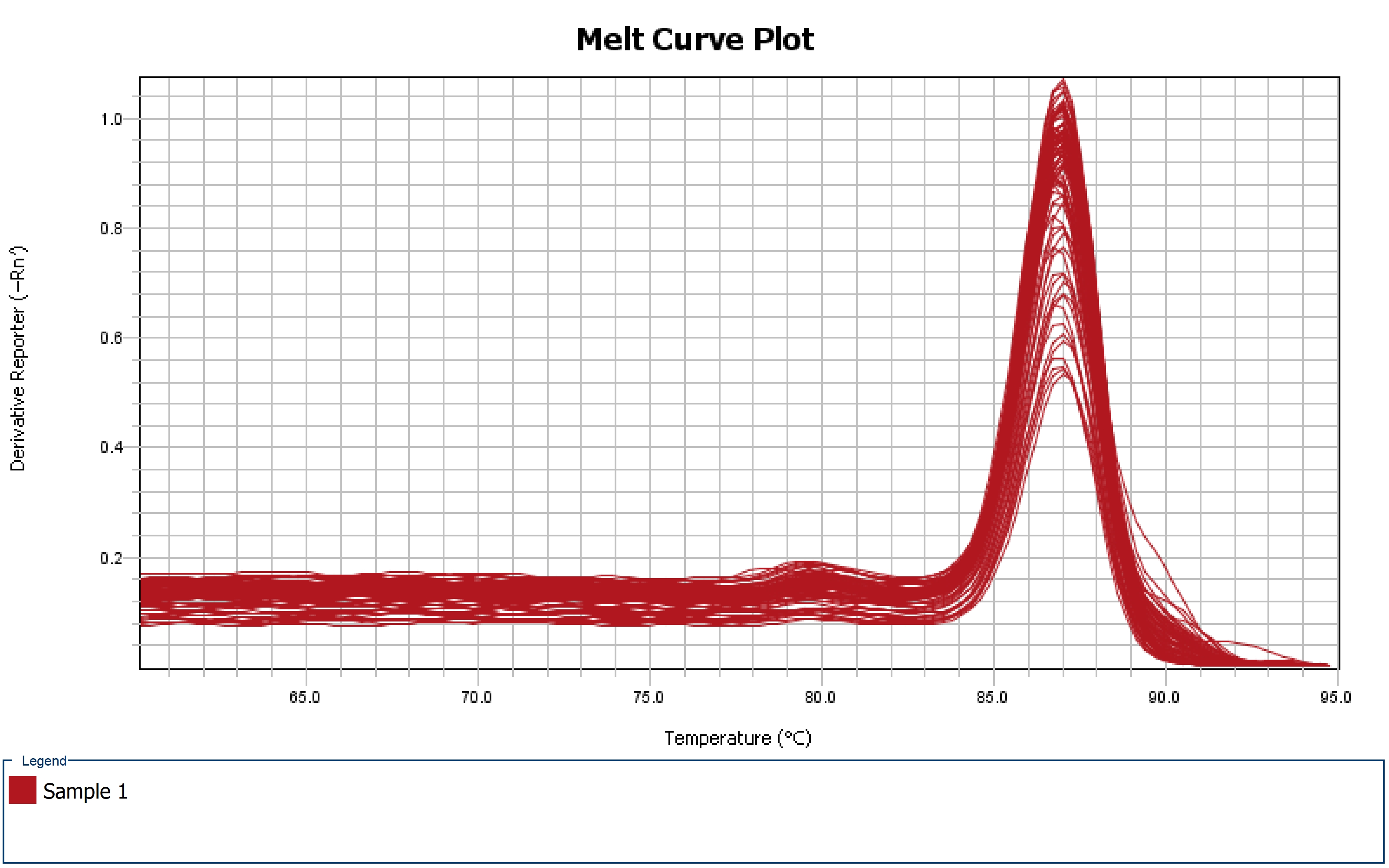

Supplement: Supplementary file 3 [file Image4.JPEG]

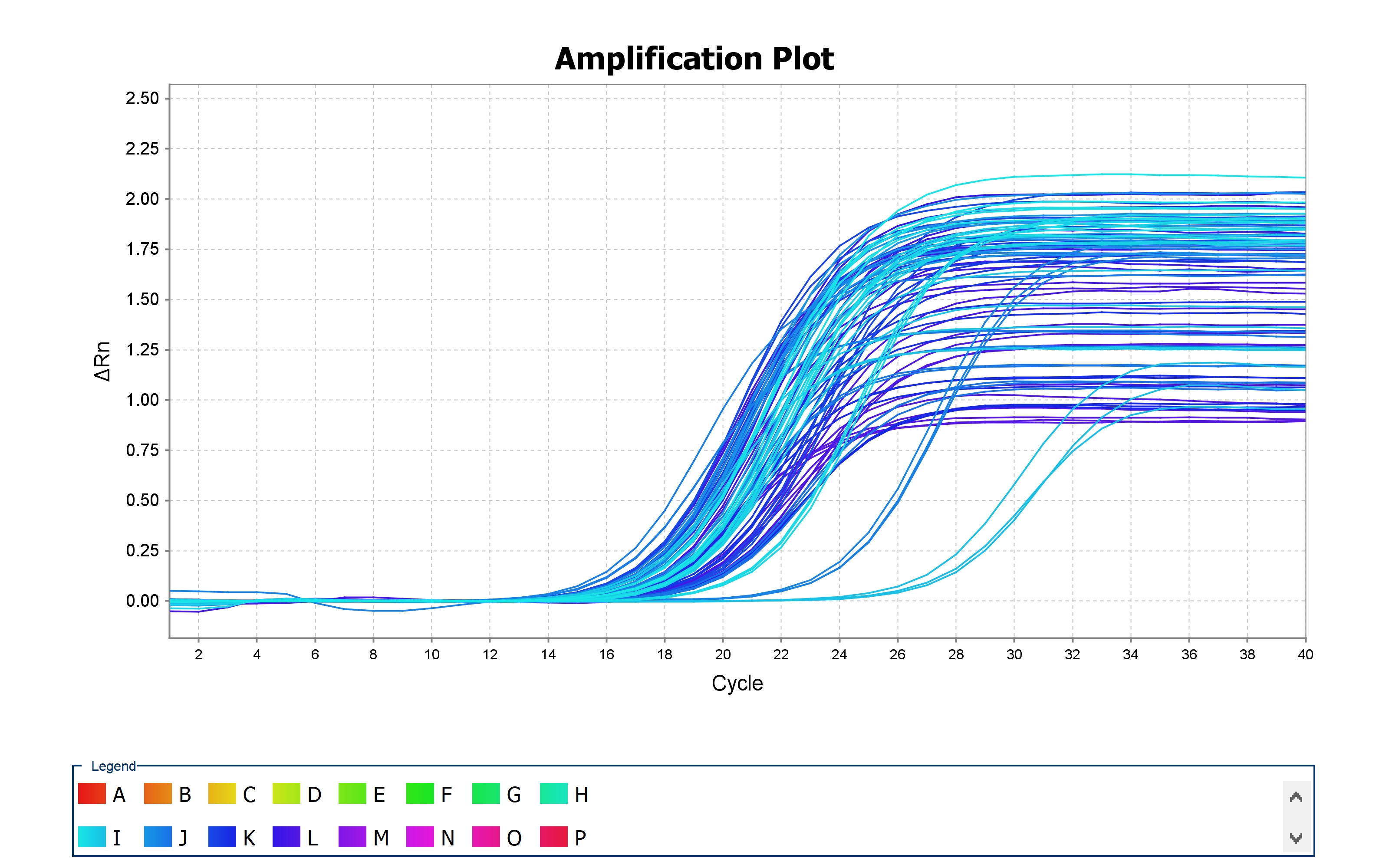

Supplement: Supplementary file 4 [file Image2.JPEG]
