# Supplementary material for: Prognostic value of SOX9 in cervical cancer: Bioinformatics and experimental approaches
Source: Front Genet. 2022 Aug 8;13:939328. doi: 10.3389/fgene.2022.939328 (PMC9394184; doi:10.3389/fgene.2022.939328)
Supplement: Supplementary file 5 [file Table2.docx]

#===================================================================

==================

#

# ⼀.DataInput

#

#===================================================================

==================

#=========================================================

# 1.数据准备⼯作(EXCEL)

#=========================================================

#准备⼯作：表观数据要把值都改为数值；在EXCEL中处理删除不需要的表观数据；

#不需要提前匹配，因为样本经聚类后需要删除离群样本

#

source("http://bioconductor.org/biocLite.R")

biocLite("WGCNA")#装包

biocLite("stringr")

#"goodSamplesGenes"与"hclust"都在WGCNA包中

#=========================================================

# 2.读取基因表达⽂件，筛选纳⼊分析的基因，评估

#=========================================================

#设置路径

setwd('C:\\Users\\zch\\Desktop\\WGCNA')

# The following setting is important, do not omit.

options(stringsAsFactors = FALSE);

#读取表达矩阵⽂件

expro=read.csv('expr.txt',sep = '\t',row.names = 1)

dim(expro)

##数据读取完成，⽅差较⼤的那些基因（意味着在不同样本中变化较⼤）

#选择了⽅差⼤于所有⽅差四分位数的基因（四分之⼀）

m.vars=apply(expro,1,var)

expro.upper=expro[which(m.vars>quantile(m.vars, probs = seq(0, 1,

0.25))[4]),]

dim(expro.upper)

write.table(expro.upper,file="geneInput_variancetop0.25.txt",sep='\t

',quote=F,row.names=T)

#通过上述步骤拿到了1825个基因的表达谱作为WGCNA的输⼊数据集，

datExpr0=as.data.frame(t(expro.upper));#转置表达矩阵

library(WGCNA)

#评估矩阵信息是否合格

gsg = goodSamplesGenes(datExpr0, verbose = 3);

gsg$allOK

#optional:当gsg不是allOK时

if (!gsg$allOK)

{

# Optionally, print the gene and sample names that were removed:

if (sum(!gsg$goodGenes)>0) printFlush(paste("Removing genes:", paste(names(datExpr0)[!

gsg$goodGenes], collapse = ", ")));

if (sum(!gsg$goodSamples)>0)

printFlush(paste("Removing samples:", paste(rownames(datExpr0)[!

gsg$goodSamples], collapse = ", ")));

# Remove the offending genes and samples from the data:

datExpr0 = datExpr0[gsg$goodSamples, gsg$goodGenes]

}

#=========================================================

# 3.样本聚类树，剔除离群样本（Sample clustering to detect outliers）

#=========================================================

#样本做聚类树

sampleTree = hclust(dist(datExpr0), method = "average");

# Plot the sample tree: Open a graphic output window of size 12 by 9

inches

# The user should change the dimensions if the window is too large

or too small.

sizeGrWindow(12,9)

#pdf(file = "Plots/sampleClustering.pdf", width = 12, height = 9);

par(cex = 0.45);

par(mar = c(0,4,2,0))

plot(sampleTree, main = "Sample clustering to detect outliers",

sub="", xlab="",

cex.lab = 1.5, cex.axis = 1.5, cex.main = 2)

#datExpr0是初始样本，datExpr是删减离群样本后

# Plot a line to show the cut

abline(h = 5000000, col = "red");

# Determine cluster under the line

clust = cutreeStatic(sampleTree, cutHeight = 5000000, minSize = 10)

table(clust)

# clust 1 contains the samples we want to keep.

keepSamples = (clust==1)

datExpr = datExpr0[keepSamples, ]

nGenes = ncol(datExpr)

nSamples = nrow(datExpr)

#=========================================================

# 4.读取，清洗表观数据，样本与表达矩阵相匹配

#=========================================================

#Input（注意此处不把第⼀列作为⾏名）

traitData = read.csv('ClinicalTraits.txt',sep = '\t');

dim(traitData)

names(traitData)

# Form a data frame analogous to expression data that will hold the

clinical traits.

#将两个样本名格式调成⼀致

library(stringr)

#traitData$sampleID <- str_replace_all(traitData$sample,'-','.');# rownames(datExpr) <- str_replace_all(rownames(datExpr),'.01','');#

删除datExpr⾏名中的.01

tumorSamples = rownames(datExpr);

#基因和表观数据的样本重新匹配（之前样本删减了部分）

traitRows = match(tumorSamples, traitData$id);

#⽤匹配的样本重组数据

datTraits = traitData[traitRows, -1];

#⾏名换为样本名

rownames(datTraits) = traitData[traitRows, 1];

#清除

collectGarbage();

#=========================================================

# 5.重建样本聚类树（Sample dendrogram and trait heatmap）

#=========================================================

# Re-cluster samples

sampleTree2 = hclust(dist(datExpr), method = "average")#再次聚类

# Convert traits to a color representation: white means low, red

means high, grey means missing entry

datTraitsColor <- numbers2colors(datTraits, signed = FALSE);#将表观数

据转为颜⾊

# Plot the sample dendrogram and the colors underneath.

sizeGrWindow(12,9)

plotDendroAndColors(sampleTree2, datTraitsColor,

groupLabels = names(datTraits),

colorHeight = 0.2,

colorHeightBase = 0.2,

colorHeightMax = 0.4,#性状部分的⾼度

rowWidths = NULL,

dendroLabels = NULL,

addGuide = FALSE, guideAll = FALSE,

guideCount = 50, guideHang = 0.2,

addTextGuide = FALSE,

cex.colorLabels = 0.8,#性状字体⼤⼩

cex.dendroLabels = 0.7, #样本聚类树中样本名字体⼤⼩

cex.rowText = 0.8,

marAll = c(1, 5, 3, 1), saveMar = TRUE,

main = "Sample dendrogram and trait heatmap")

save(datExpr, datTraits,file = "G-01-dataInput.RData")

#=====================================================================================

#

# ⼆.network construt---three methods

#

#===================================================================

==================

# Load the WGCNA package

library(WGCNA)

# The following setting is important, do not omit.

options(stringsAsFactors = FALSE);

# Allow multi-threading within WGCNA. At present this call is

necessary.

# Any error here may be ignored but you may want to update WGCNA if

you see one.

# Caution: skip this line if you run RStudio or other third-party R

environments.

# See note above.

enableWGCNAThreads()

# Load the data saved in the first part

lnames = load(file = "G-01-dataInput.RData");

#The variable lnames contains the names of loaded variables.

lnames

#=========================================================

# 1.选择合适的软阈值

#=========================================================

# Choose a set of soft-thresholding powers

powers = c(c(1:10), seq(from = 12, to=20, by=2))

# Call the network topology analysis function

sft = pickSoftThreshold(datExpr, powerVector = powers, verbose = 5)#

确定软阈值

# Plot the results:

sizeGrWindow(9, 5)

par(mfrow = c(1,2));

cex1 = 0.9;

# Scale-free topology fit index as a function of the soft

thresholding power

plot(sft$fitIndices[,1], -sign(sft$fitIndices[,3])*sft$fitIndices[,

2],

xlab="Soft Threshold (power)",ylab="Scale Free Topology Model

Fit,signed R^2",type="n",

main = paste("Scale independence"));

text(sft$fitIndices[,1], -sign(sft$fitIndices[,3])*sft$fitIndices[,

2],

labels=powers,cex=cex1,col="red");

# this line corresponds to using an R^2 cut-off of h

abline(h=0.90,col="red")

#平均连接度

# Mean connectivity as a function of the soft-thresholding power

plot(sft$fitIndices[,1], sft$fitIndices[,5],

xlab="Soft Threshold (power)",ylab="Mean Connectivity",

type="n",

main = paste("Mean connectivity"))text(sft$fitIndices[,1], sft$fitIndices[,5], labels=powers,

cex=cex1,col="red")

# here we define the adjacency matrix using soft thresholding with

beta=6

ADJ1=abs(cor(datExpr,use="p"))^5

# When you have relatively few genes (<5000) use the following code

k=as.vector(apply(ADJ1,2,sum, na.rm=T))#⼆选⼀即可

# When you have a lot of genes use the following code

k=softConnectivity(datE=datExpr,power=5)

# Plot a histogram of k and a scale free topology plot

sizeGrWindow(10,5)

par(mfrow=c(1,2))

hist(k)

scaleFreePlot(k, main="Check scale free topology\n")

#=========================================================

# 2.转为邻接矩阵（幂次）

#=========================================================

softPower = 5;

adjacency = adjacency(datExpr, power = softPower)

#=========================================================

# 3.转为拓扑矩阵，计算相异度dissTOM

#=========================================================

TOM = TOMsimilarity(adjacency);#计算时间很⻓，公式⽐较复杂

dissTOM = 1-TOM

#=========================================================

# 4.分层聚类，画聚类树（Gene clustering on TOM-based dissimilarity）

#=========================================================

# ⽤相异度dissTOM进⾏聚类

geneTree = hclust(as.dist(dissTOM), method = "average");

# Plot the resulting clustering tree (dendrogram)

sizeGrWindow(12,12)

plot(geneTree, xlab="", sub="", main = "Gene clustering on TOM-based

dissimilarity",

labels = FALSE, hang = 0.04);

#=========================================================

# 5.确定模块，动态树剪切，定义颜⾊ （Gene dendrogram and module colors）

#=========================================================

# 模块⾄少含30个基因（较⼤模块相对更有意义）

minModuleSize = 30;

# Module identification using dynamic tree cut:

dynamicMods = cutreeDynamic(dendro = geneTree, distM = dissTOM,

deepSplit = 2, pamRespectsDendro =

FALSE,

minClusterSize = minModuleSize);

table(dynamicMods)#各模块信息# 将模块序号转为颜⾊

dynamicColors = labels2colors(dynamicMods)

table(dynamicColors)

# 聚类树和模块信息整合，画图

sizeGrWindow(8,12)

plotDendroAndColors(geneTree, dynamicColors, "Dynamic Tree Cut",

dendroLabels = FALSE, hang = 0.03,

addGuide = TRUE, guideHang = 0.05,

main = "Gene dendrogram and module colors")

#===================================================================

==================

# 6.计算eigengene，对模块进⾏分层聚类，合并较为相似的模块

#===================================================================

==================

# 计算 eigengenes

MEList = moduleEigengenes(datExpr, colors = dynamicColors)

MEs = MEList$eigengenes#MEs为每个模块对应每个样本的eigengene（是⼀个值）

# Calculate dissimilarity of module eigengenes

MEDiss = 1-cor(MEs);#先计算MEs之间的相关度，然后计算MEs的相异度

# Cluster module eigengenes，以相异度聚类

METree = hclust(as.dist(MEDiss), method = "average");

# Plot the result

sizeGrWindow(7, 6)

plot(METree, main = "Clustering of module eigengenes",

xlab = "", sub = "")

#===================================================================

==================

# 7.设置abline=0.25，将聚类树上相似的模块合并

#===================================================================

==================

MEDissThres = 0.25

# 划线abline=0.25

abline(h=MEDissThres, col = "red")

# 合并相似模块函数

merge = mergeCloseModules(datExpr, dynamicColors, cutHeight =

MEDissThres, verbose = 3)

# The merged module colors

mergedColors = merge$colors;

# Eigengenes of the new merged modules:

mergedMEs = merge$newMEs;

#=====================================================================================

# 8.⽤剪切后的模块，画新的聚类树和模块图

#===================================================================

==================

sizeGrWindow(12, 9)

#pdf(file = "Plots/geneDendro-3.pdf", wi = 9, he = 6)

plotDendroAndColors(geneTree, cbind(dynamicColors, mergedColors),

c("Dynamic Tree Cut", "Merged dynamic"),

dendroLabels = FALSE, hang = 0.03,

addGuide = TRUE, guideHang = 0.05)

#dev.off()

#===================================================================

==================

# 9.重命名⼯作,保存

#===================================================================

==================

# Rename to moduleColors

moduleColors = mergedColors

# 加⼊grey的颜⾊序号

colorOrder = c("grey", standardColors(50));

moduleLabels = match(moduleColors, colorOrder)-1;

MEs = mergedMEs;

# Save module colors and labels for use in subsequent parts

save(MEs, moduleLabels, moduleColors, geneTree, file = "G-02-

networkConstruction-StepByStep.RData")

#===================================================================

==================

#

# 三。模块结合表观数据（relateModsToEXt）

#

#===================================================================

==================

#===========================================================

# 1.读取之前的数据

#===========================================================

# Load the WGCNA package

library(WGCNA)

# The following setting is important, do not omit.

options(stringsAsFactors = FALSE);

# Load the expression and trait data saved in the first part

lnames = load(file = "G-01-dataInput.RData");

#The variable lnames contains the names of loaded variables.

lnames

# Load network data saved in the second part.

lnames = load(file = "G-02-networkConstruction-StepByStep.RData");lnames

#===========================================================

# 2.计算模块与表观数据的相关性

#===========================================================

# Define numbers of genes and samples

nGenes = ncol(datExpr);#定义基因和样本的数量

nSamples = nrow(datExpr);

# 计算之前合并得到的新模块的eigengenes

MEs0 = moduleEigengenes(datExpr, moduleColors)$eigengenes

#将MEs⽤MEs0的顺序排列

MEs = orderMEs(MEs0)

#计算模块与表观数据的相关性

moduleTraitCor = cor(MEs, datTraits, use = "p");

moduleTraitPvalue = corPvalueStudent(moduleTraitCor, nSamples);

#画模块内基因表达热图，特征向量柱形图

#热图

datExpr_log<-log10(datExpr+0.00001)

which.module="green"

sizeGrWindow(8,7)

ME=MEs[,paste("ME",which.module,sep='')]

par(mfrow=c(2,1),mar=c(0.3,5.5,3,2))

plotMat(t(scale(datExpr_log[,moduleColors==which.module])),nrgcols=3

0,rlabels=T,

rcols=which.module,main=which.module,cex.main=2)

#柱形图 eigengene expression plot

par(mar=c(5,4.2,2,0.7))

barplot(ME,col=which.module,main="",cex.main=2,ylab="eigengene

expression",xlab="")

#导出ME

#row.names(MEs0)=row.names(datExpr)

#write.table(MEs0,file="MEs0.txt",sep='\t',quote=F,row.names=T)

#===========================================================

# 3.作图（Module-trait relationships）

#===========================================================

sizeGrWindow(10,8)

# Will display correlations and their p-values

textMatrix = paste(signif(moduleTraitCor, 2), "\n(",

signif(moduleTraitPvalue, 1), ")", sep = "");

dim(textMatrix) = dim(moduleTraitCor)

par(mar = c(9, 8.5, 3, 3));

# Display the correlation values within a heatmap plot

labeledHeatmap(Matrix = moduleTraitCor,

xLabels = names(datTraits), yLabels = names(MEs),

ySymbols = names(MEs),

colorLabels = FALSE,

colors = greenWhiteRed(50),

textMatrix = textMatrix,

setStdMargins = FALSE,

cex.text = 0.5,

zlim = c(-1,1),

main = paste("Module-trait relationships"))

#==============================================================

# 4.选择感兴趣的Trait进⼀步计算geneModuleMembership，

geneTraitSignificance

#==============================================================

# Define variable futime containing the futime column of datTrait

Surtime = as.data.frame(datTraits$futime);

names(Surtime) = "Surtime"

# names (colors) of the modules

modNames = substring(names(MEs), 3)

#计算geneModuleMembership和MMPvalue

geneModuleMembership = as.data.frame(cor(datExpr, MEs, use = "p"));

MMPvalue =

as.data.frame(corPvalueStudent(as.matrix(geneModuleMembership),

nSamples));

#在列名上加MM，p.MM

names(geneModuleMembership) = paste("MM", modNames, sep="");

names(MMPvalue) = paste("p.MM", modNames, sep="");

#计算某项Trait（ER）中的GS(genesignificance)

geneTraitSignificance = as.data.frame(cor(datExpr, Surtime, use =

"p"));

GSPvalue =

as.data.frame(corPvalueStudent(as.matrix(geneTraitSignificance),

nSamples));

names(geneTraitSignificance) = paste("GS.", names(Surtime), sep="");

names(GSPvalue) = paste("p.GS.", names(Surtime), sep="");

#==========================================================

# 5.选择模块，作模块membership和genesignificance的相关图

#==========================================================

#选择相关性⾼的模块

module = "turquoise"

column = match(module, modNames);

moduleGenes = moduleColors==module;

sizeGrWindow(7, 7);

par(mfrow = c(1,1));

verboseScatterplot(abs(geneModuleMembership[moduleGenes, column]),

abs(geneTraitSignificance[moduleGenes, 1]), xlab = paste("Module Membership in", module,

"module"),

ylab = "Gene significance for Surtime",

main = paste("Module membership vs. gene

significance\n"),

cex.main = 1.2, cex.lab = 1.2, cex.axis = 1.2,

col = module)

#==========================================================

# 6.探针名转为基因名

#==========================================================

#显示基因or探针名

names(datExpr)

#显示blue模块中的基因名

names(datExpr)[moduleColors=="turquoise"]

#探针match genesymbol

#annot = read.csv(file = "GeneAnnotation.csv");

#dim(annot)

#names(annot)

#probes = names(datExpr)

#probes2annot = match(probes, annot$substanceBXH)

# The following is the number or probes without annotation:

#sum(is.na(probes2annot))

# Should return 0.

#===================================================================

# 9.创建基因信息表（含每个基因对应的genesymbol,color,GS,p.GS,MM）

#===================================================================

# Create the starting data frame

geneInfo0 = data.frame(geneSymbol = rownames(geneTraitSignificance),

#创建geneinformation的frame，含

genesymbol,color，GS，MM等

moduleColor = moduleColors,

geneTraitSignificance,

GSPvalue)

# Order modules by their significance for ER

modOrder = order(-abs(cor(MEs, Surtime, use = "p")));

# Add module membership information in the chosen order

for (mod in 1:ncol(geneModuleMembership))#在上⾯的表格中加⼊MM的信息

{

oldNames = names(geneInfo0)

geneInfo0 = data.frame(geneInfo0, geneModuleMembership[,

modOrder[mod]],

MMPvalue[, modOrder[mod]]);

names(geneInfo0) = c(oldNames, paste("MM.",

modNames[modOrder[mod]], sep=""),

paste("p.MM.", modNames[modOrder[mod]],

sep=""))

}# Order the genes in the geneInfo variable first by module color,

then by geneTraitSignificance

geneOrder = order(geneInfo0$moduleColor, -

abs(geneInfo0$GS.Surtime));

geneInfo = geneInfo0[geneOrder, ]

#=======================================================

# 10.保存

#=======================================================

write.csv(geneInfo, file = "geneInfo.csv")

##########################

#### 计算模块内连接度###

#########################

# Select module

module = "turquoise";

# Select module probes

probes = names(datExpr)

inModule = (moduleColors==module);

modProbes = probes[inModule];

IMConn = softConnectivity(datExpr[, modProbes],power=5);

dat1=datExpr[inModule]

datExp_IMConn <-data.frame(IMConn,t(dat1))

datExp_IMConn=data.frame(datExp_IMConn)

write.table(datExp_IMConn,

file =

paste("Intramodule_connectivity-",module," .txt"),sep='\t')

#计算In Moudel的连接度，选择top30

nTop = 30;

top = (rank(-IMConn) <= nTop)#选择连接度最⾼的30个基因

dat2=t(datExp_IMConn)

dat2<-data.frame(dat2)

dat3<-dat2[top]

dat3<-t(dat3)

dat3<-data.frame(dat3)

write.table(dat3,

file = paste("Intramodule_connectivity-",module,"-

top30.txt"),sep='\t')

#===================================================================

==================

# 五。结果可视化

# 1.数据读取

#================================================

setwd('C:\\Users\\PYB\\Desktop\\wgcna_GBM\\WGCNA\\WGCNA');

# Load the WGCNA packagelibrary(WGCNA)

# The following setting is important, do not omit.

options(stringsAsFactors = FALSE);

# Load the expression and trait data saved in the first part

lnames = load(file = "G-01-dataInput.RData");

#The variable lnames contains the names of loaded variables.

lnames

# Load network data saved in the second part.

lnames = load(file = "G-02-networkConstruction-StepByStep.RData");

lnames

nGenes = ncol(datExpr)

nSamples = nrow(datExpr)

#================================================

# 2。计算dissTOM，基于拓扑重叠做基因⽹络热图

#================================================

# Calculate topological overlap anew: this could be done more

efficiently by saving the TOM

# calculated during module detection, but let us do it again here.

dissTOM = 1-TOMsimilarityFromExpr(datExpr, power = 5);#重新计算基因之

间的dissTOM

# Transform dissTOM with a power to make moderately strong

connections more visible in the heatmap

plotTOM = dissTOM^5;#dissTOM幂次计算，使热图更加明显

# Set diagonal to NA for a nicer plot

diag(plotTOM) = NA;#设置斜对⻆线为NA

# Call the plot function

sizeGrWindow(9,9)

TOMplot(plotTOM, geneTree, moduleColors, main = "Network heatmap

plot, all genes")

#热图每⾏及每列对应⼀个基因，颜⾊越深表示拓扑重叠越⾼，基因之间的紧密度也越⾼

#===================================================================

==================

# 3.随机挑选基因进⾏验证???

#===================================================================

==================

nSelect = 1000

# For reproducibility, we set the random seed

set.seed(10);

select = sample(nGenes, size = nSelect);

selectTOM = dissTOM[select, select];

# There's no simple way of restricting a clustering tree to a subset

of genes, so we must re-cluster.

selectTree = hclust(as.dist(selectTOM), method = "average")

selectColors = moduleColors[select];

# Open a graphical window

sizeGrWindow(9,9)# Taking the dissimilarity to a power, say 10, makes the plot more

informative by effectively changing

# the color palette; setting the diagonal to NA also improves the

clarity of the plot

plotDiss = selectTOM^5;

diag(plotDiss) = NA;

TOMplot(plotDiss, selectTree, selectColors, main = "Network heatmap

plot, selected genes")

#===================================================================

==================

# 4.将表观数据纳⼊ME，统⼀制作ME相关性的热图

#===================================================================

==================

# 重新计算模块MEs

MEs = moduleEigengenes(datExpr, moduleColors)$eigengenes

# 从表观数据中分离Surtime

Surtime = as.data.frame(datTraits$futime);

names(Surtime) = "Surtime"

# Surtime加⼊MEs成为MET

MET = orderMEs(cbind(MEs, Surtime))

# Plot the relationships among the eigengenes and the trait

sizeGrWindow(5,7.5);

par(cex = 0.9)

plotEigengeneNetworks(MET, "", marDendro = c(0,4,1,5), marHeatmap =

c(2,4,1,2), cex.lab = 0.8,

xLabelsAngle = 90)#聚类树和热图结

# 单独画聚类图

sizeGrWindow(6,6);

par(cex = 1.0)

plotEigengeneNetworks(MET, "Eigengene dendrogram", marDendro =

c(0,4,2,0),

plotHeatmaps = FALSE)

# 单独画热图

par(cex = 1.0)

plotEigengeneNetworks(MET, "Eigengene adjacency heatmap", marHeatmap

= c(3,4,2,2),

plotDendrograms = FALSE, xLabelsAngle = 90)

#===================================================================

==================

#

# 六。

#

#===================================================================

==================

#================================================# 1.数据读取

#================================================

setwd('C:\\Users\\PYB\\Desktop\\wgcna_GBM\

\WGCNA_variance0.25_stepbystep\\WGCNA');

# Load the WGCNA package

library(WGCNA)

# The following setting is important, do not omit.

options(stringsAsFactors = FALSE);

# Load the expression and trait data saved in the first part

lnames = load(file = "GBM-01-dataInput.RData");

#The variable lnames contains the names of loaded variables.

lnames

# Load network data saved in the second part.

lnames = load(file = "GBM-02-networkConstruction-StepByStep.RData");

lnames

#==================================================================

# 2.计算感兴趣模块中基因之间的拓扑重叠（为相关性)，导出cytoscape可以识别的⽂

件，然后在cytoscape中作图

#==================================================================

# Recalculate topological overlap if needed

TOM = TOMsimilarityFromExpr(datExpr, power = 5);

# Select mbodules

modules = c("turquoise");

# Select module probes

probes = names(datExpr)

inModule = is.finite(match(moduleColors, modules));

modProbes = probes[inModule];

modGenes = modProbes;

# Select the corresponding Topological Overlap

modTOM = TOM[inModule, inModule];

dimnames(modTOM) = list(modProbes, modProbes)

#===================================================================

==================

# 3.选择模块中连接度最⾼的30个基因，计算拓扑重叠，导出txt⽂件

#===================================================================

==================

nTop = 30;

#计算In Moudel的连接度，选择top30

IMConn = softConnectivity(datExpr[, modProbes]);

top = (rank(-IMConn) <= nTop)#选择连接度最⾼的30个基因

#计算30个基因之间之间的拓扑重叠，导出为txt⽂件

# Export the network into edge and node list files Cytoscape can

readcyt = exportNetworkToCytoscape(modTOM[top, top],

edgeFile = paste("CytoscapeInput

edges-", paste(modules, collapse="-"), ".txt", sep=""),

nodeFile = paste("CytoscapeInput

nodes-", paste(modules, collapse="-"), ".txt", sep=""),

weighted = TRUE,

threshold = 0.02,

nodeNames = modProbes,

altNodeNames = modGenes,

nodeAttr = moduleColors[inModule])
